# Using constraints and their value for optimization of large ODE systems

**DOI:** 10.1098/rsif.2014.1303

**Published:** 2015-03-06

**Authors:** Mirela Domijan, David A. Rand

**Affiliations:** 1 Sainsbury Laboratory, University of Cambridge, Cambridge CB2 1LR, UK; 2 Warwick Systems Biology Centre and Mathematics Institute, University of Warwick, Coventry CV4 7AL, UK

**Keywords:** systems biology models, experimental optimization, statistical estimation, circadian clocks, signalling systems

## Abstract

We provide analytical tools to facilitate a rigorous assessment of the quality and value of the fit of a complex model to data. We use this to provide approaches to model fitting, parameter estimation, the design of optimization functions and experimental optimization. This is in the context where multiple constraints are used to select or optimize a large model defined by differential equations. We illustrate the approach using models of circadian clocks and the NF-*κ*B signalling system.

## 1. Introduction

Systems Biology is producing a rapidly growing number of complex mathematical models of dynamic biological systems such as the cell cycle, circadian clocks and numerous signalling systems. These models are usually highly nonlinear and have many state variables and parameters. It is therefore very challenging to understand how the behaviour of these models depends upon model structure and parameters and to distinguish those features of the model that are fundamental from those that are accidental or irrelevant. Moreover, the nonlinearity and large size of these models makes validation and calibration against biological data very difficult.

We focus in this paper on large models given by differential equations, the most ubiquitous method for such systems. When estimating the parameters of such systems, it is usual to either introduce a likelihood expressing the probability of the data given a particular deterministic solution or provide a function measuring the fit of such a solution to the data. A common choice for a likelihood is to assume that intrinsic noise can be neglected and that the main source of stochasticity is observational error, which is often assumed to be normally distributed. Optimization functions are often based on the Euclidean distance or are a sum of squares each measuring the deviation of a summary statistic from the data derived value. For the most complex models, it is often the case that the data only partially constrain the model and therefore, for these models, such fitting is done by hand or using optimization functions, where the modellers have to identify qualitative features of interest.

In each case, there is a great need for analytical tools to facilitate fitting and to provide a rigorous assessment of the quality and value of the fit and our aim here is to provide some mathematical tools to tackle both of these challenges. To demonstrate the usefulness of these tools, we apply them to some significant exemplar models. We are particularly interested in large models with many parameters and state variables. For example, one of the models we consider has 28 state variables and 104 parameters and as we combine this with models for some mutants, the effective number of state variables is several times this.

Suppose that we are considering such a system and that we have data and models for the wild-type and a number of mutants in a set of conditions. For example, the data might be for a wild-type circadian clock or signalling system and several gene knockouts in a number of environmental conditions. We assume that for each such combination of genetic background and environmental conditions (which we henceforth call GE-combinations), we have a model and that from the data we have a set of constraints that the models should satisfy. Note that in this paper, when we talk of the constraints, we mean quantitative properties usually derived from experimental data. They might, for example, quantify the levels of certain mRNAs or proteins for the given combination, or in the case of oscillating systems they might determine the period of the oscillation or the relative phases of the mRNAs or proteins. In general, they will be of the form 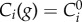 for some real-valued function *C_i_* of the solution *g* of interest for one of the given GE-combinations. The value of 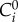 will come from the experimental data. As usual it is just the parameters *k* that we are changing, *g*, and hence *C_i_*, is a real-valued function of them and the conditions to be satisfied are of the form 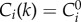. A constraint determines the value of some quantity.

Often these constraints are collected into a single real-valued function to be locally optimized which effectively acts as a likelihood function. Approximate Bayesian computation (ABC) functions in this way. ABC methods seek to infer parameters by comparing simulated data to the observed data, in terms of an optimization function that combines a set of summaries *C* = (*C*_1_, *…* , *C_m_*) essentially equivalent to the constraints mentioned above [1–3]. A related, less sophisticated approach that has been successfully employed is to search subspaces of the space of parameters using such an optimization function to find approximate local optima [4,5]. For both sorts of methods, the function to be optimized is usually of the form
1.1
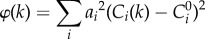

and a key question that we discuss below is how to choose the function, for example, in the case of (1.1), what constraints *C_i_* should be used and how should their weights *a_i_* be chosen.

However, in the main, our approach will be to consider the optimization problem in terms of a set of *m* individual constraints rather than to try and incorporate them into a single function to be optimized. We consider this in the context of a combined model for a set of GE-combinations as formulated below. The theory that we present allows us to investigate a number of interesting aspects. Firstly, we can consider a combined model that satisfies a set of constraints *C*_1_, *…* , *C_m_* and gives a quantitative measure of the extent to which the constraints actually constrain the model. Given that a set of constraints have been applied, we quantify the extent to which an additional constraint *C_m_*_+1_ further constrains the model and explain how its constraint value can be measured. Secondly, we show that it will usually be the case that the constraint value declines very rapidly as *m* increases. In fact, we also demonstrate that it is reasonable to expect that many of the constraints will have small norm and hence be ineffective. Thirdly, we prove a theorem (called *m → m* + 1 Transition theorem) that allows us to use this constraint value to determine the effects of adding a new constraint to the form of the optimization problem both in terms of geometry, analysis and stochastic optimization. Fourthly, we consider the construction of optimization functions and show how constraint value can be used to help design them for use in statistical estimation algorithms. Finally, we discuss how to use the results for experimental design. We want to facilitate the choice of effective constraints that will better characterize the system when deciding what experiments to do. We give some examples of how this can be done using our framework.

It is important to stress at this point that we are not providing an algorithm for estimating parameter values. However, as we provide an approach to analytically determine which sets of constraints are most informative, it can be used to help determine which are the most useful to use in some estimation algorithms.

In general, the constraints *C_i_* of interest are nonlinear. Unfortunately, a general global nonlinear theory is not possible because our current understanding of dynamical systems, though extensive, is not adequate for this. However, we can develop a relatively powerful and useful theory based on local analysis about a particular set of parameter values. This uses the extensive and powerful perturbation theory for differential equations.

## 2. Mathematical preliminaries

We assume that we have a set of models described by a system of differential equations of the form 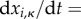 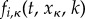. Each model is for a given GE-combination *κ*. Here, *t* is time and the vector *x_κ_* = (*x*_1,_*_κ_*, *…*, *x_n,κ_*) represents the state variables (typically for our applications, mRNA and protein levels). For this GE-combination *κ*, we can write this system as
2.1
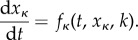



There is a common vector of parameters *k* = (*k*_1_, *…* , *k_s_*) for all such models. We then integrate all these models into a single one given by
2.2
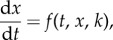

where *t* is time, 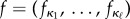 and 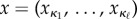, where 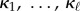 is the set of GE-combinations being considered.

We also assume that for each of the systems (2.1), d*x*_*k*_/d*t* = *f*_*k*_ (*t*, *x*_*k*_, *k*) there is a solution *x_*κ*_* = *g_*κ*_*(*t*, *k*) or a class of solutions defined for a specific time range 0 ≤ *t* ≤ *T_*κ*_* that are of particular interest. For example, for circadian oscillations, the primary object of interest is an attracting periodic orbit of equation (2.1) and *T_*κ*_* will be the period of this orbit. On the other hand, for models of signalling systems, one is often interested in a solution that is not periodic but is defined by a given initial condition *x*_0_. Such a signalling system is usually also subject to a given perturbation caused by an incoming signal and this will typically be modelled by a sudden change in a system parameter or by the time dependence of the right-hand side of equation (2.1).

In regulatory and signalling systems, the values of two parameters may differ by an order of magnitude or more. Therefore, it is usually not appropriate to consider absolute changes in the parameters *k_j_*, but instead to consider relative changes. A good way to do this is to introduce new parameters 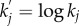 because absolute changes in 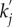 correspond to relative changes in *k_j_*. Then for small changes *δk_j_* to the parameters, the corresponding change to 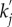 is 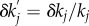 which is scaled and non-dimensional. We adopt the convention that our non-zero parameters 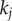 are henceforth these logged parameters 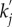. In fact, the theory applies equally well to the unscaled parameters but in our examples we always use logged parameters for the reason given above.

We are interested in the size of the variation of a constraint as parameters are varied. However, in the biological problems we are interested in the optimal value 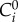 of a constraint *C_i_* is determined by data and this is always only estimated. Therefore, we need to take account of the standard error 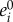 (i.e. the estimated standard deviation of the mean) of the experimentally observed values of 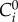. The constraint *C_i_* is not effective if the variation is small compared with 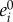. For our presentation, it is convenient to always normalize the constraints by replacing *C_i_* by 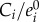. Therefore, in what follows, by a constraint, we always mean one that has been normalized by its standard error of the corresponding data.

### 2.1. Constraints and their value

We firstly consider the behaviour of the constraints about a parameter value *k* = *k*_∗_. According to Taylor's theorem, the local variation of *C_i_*(*k*) about *k* = *k*_∗_ is given by
2.3
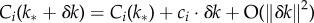

where *c_i_* = (*c_i_*_,1_, *…* , *c_i_*_,*s*_) is the derivative of *C_i_* at *k*_∗_ and · denotes the usual dot product between vectors. Thus, 
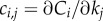
 evaluated at *k*_∗_. We therefore call the vectors *c_i_* in 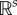 the *linearized constraints*.

If we have a set of constraints 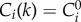, *i* = 1, *…* , *m*, with associated linear constraints *c_i_* at *k* = *k*_∗_, there are two ways in which they can be ineffective. Firstly, such a constraint *C_i_* might be insensitive to variation in the parameters at *k*_∗_ which means that *c_i_* will have small norm 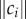 where 
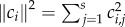
. This is because, up to the second-order terms O(‖*δk*‖^2^),

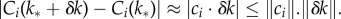



**Figure 1.**
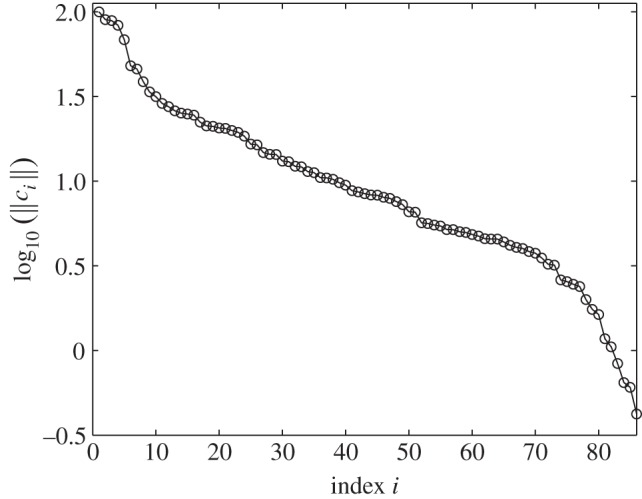
Norms of the scaled Pokhilko model constraints, 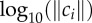, in order of decreasing value (open circles). Each norm has been scaled by dividing by the standard error of the constraint.

One might think that constraints would be chosen to ensure that they did not have a small norm. However, in figure 1, we show the norms of the constraints that were chosen for an important model of the circadian clock. We see that many have very small norms. This is not some mistake on the part of the authors but, surprisingly, is inevitable for systems like this as we explain below. Even more importantly, the theory we present explains why we should expect that large sets of constraints are often strongly non-independent.

The second way is that a linearized constraint in this set might not be very independent of the other linearized constraints in it because it is very close to being a linear combination of them. This is a problem because then this constraint will be largely determined by the others. We now give a precise description of this.

Suppose that we have such a set of linearized constraints *c*_1_, *…* , *c_m_*. For any other linearized constraint *c* = *c_m_*_+1_ define 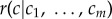 to be the unique vector orthogonal to *c*_1_, *…* , *c_m_* such that
2.4
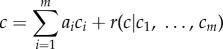



for some *a*_1_, *…* , *a_m_*.

Then for the constraint *C* to be effective when we have already applied the other constraints *C_i_*, we need that 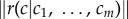 is not too small. This is because in changing *δk* = *k* − *k*_∗_ to optimize the value of *c*, the part 
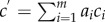
 is not allowed to change as it is determined by the other constraints *C_i_*. Therefore, only 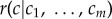 can change and as this has a small norm, the constraint *C* only has small variation around *k*_∗_.

Let us explain this in a little more detail. Suppose that the *C_i_* for *i* = 1, *…* , *m* have been optimized so that 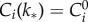. Suppose also that we now want to add the new constraint *C_m_*_+1_ for which 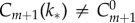 and tune *k*_∗_ to 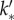 so that 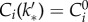 for *i* = 1, *…* , *m* + 1. Then, assuming 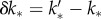 is small, from (2.3), we require that 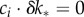. Therefore, if 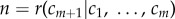, by (2.4), 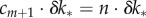 and consequently by (2.3), 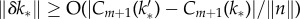. Thus, if 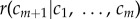 is small, the parameter change that will be needed will be much larger than the change in *C_m_*_+1_ that is required.

We therefore make the following definition.

**Definition 2.1.**

*The constraint value of a constraint C or linearized constraint c relative to a set of constraints* 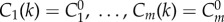 *or linearized constraints c*_1_, *…* , *c_m_ is denoted* 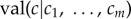 *and given by*

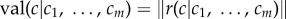

The use of the standard error for normalization was discussed above. It is also important to note that it sets a natural scale which is necessary because if the normalization is omitted, then it is possible by scaling to trivially increase the constraint value because 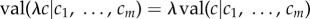 for all *λ* > 0. Importantly, its use makes the constraint value non-dimensional.

Moreover, we emphasize that the practical use of constraints such as 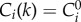 rarely requires exact values for 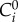. All the applications we consider only require reliable estimates of the order of magnitude of 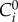. Thus only approximate determination of the standard errors 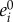 is usually required. For some of the data in the examples we discuss, standard errors were not available but we could estimate the standard deviation and therefore we approximated the standard error by this.

We will say that a set of constraints is *non-degenerate* if they are linearly independent. Many of the results that we discuss rely on an analysis of the matrix *M* = *M*(*c*_1_, *…* , *c_m_*) whose *i*th row is the vector *c_i_*. In particular, the constraints are non-degenerate if the rank of *M* is maximal, i.e. *m*.

Now suppose that for *i* = 2, *…* , *m*, 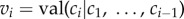 and define 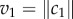. Then an important result (electronic supplementary material, theorem S2) is that one can reorder the constraints so that *v*_1_ is maximal among all such orderings and so that *v*_1_ ≥ *v*_2_ ≥ ⋯ ≥ *v_m_*. This ordering is effectively unique subject to the possibility that there might be multiple constraints with the same *v_i_*. We say that such a set of constraints is *ordered*. Electronic supplementary material, theorem S2, provides a fast way to calculate the ordering of the constraint set using the *LQ*-decomposition of a matrix.

A set of constraints can only be non-degenerate if *m* ≤ *s*. However, the result about ordering the constraint values works equally well for the case *m* > *s*. This can be thought of as an optimal choice of a subset of *s* independent constraints. The remaining constraints will be linearly dependent upon this subset.

## 3. Important exemplar systems: clocks and signals

In this section, we will demonstrate the practical use of our mathematical tools using three key examples. Our examples showcase the wide range applicability of our methodology both in terms of systems and the type of constraint.

### 3.1. Pokhilko 2012 model of the plant circadian clock

An important recent model of the plant circadian clock from [6] consists of *n* = 28 variables representing the levels of the following: mRNA and protein of the genes LHY, CCA1, TOC1, PRR9, PRR7, NI, LUX and ELF4; ZTL protein, LHY modified protein; mRNA of ELF3 and GI, cytoplasmic proteins of ELF3, GI, COP1; nuclear proteins of ELF3; GI and COP1 in day and night forms; and the cytoplasmic protein complexes ELF3-GI, GI-ZTL (ZG) and nuclear protein complexes ELF3-GI, ELF3-ELF4 and EC.

The model has a complex structure incorporating multiple positive and negative feedback loops with the interaction between components described by *s* = 104 parameters. It has been constrained by an impressively large collection of experimental data from the plant *Arabidopsis thaliana* with various genetic backgrounds and tested under different environmental conditions. Most of the parameters are fitted to the biological data or their values are taken from earlier models that were likewise fitted to data. Six of the parameters represent Hill coefficients whose values were not fitted: instead they were fixed either for the sake of simplicity or taken to correspond to the experimental evidence of protein dimerization in some of the gene interactions. We assume that these six do not form the part of the set of parameters that can be perturbed.

The model parameter fitting procedure aimed (i) to minimize the deviation of model simulated mRNAs from the normalized experimental data for nine key genes in WT plants under cycles of 12 h of light followed by 12 h of dark (denoted 12 L : 12 D) and (ii) to fit the clock oscillation period in plants in different GE-backgrounds where the plant is either in constant darkness or constant light (denoted DD or LL). The various GE-combinations and constraints are shown in table 1.

**Table 1.** The GE-combinations of the Pokhilko model [6].

GE-comb.	genetic background	entraining signal	constraints
*κ*_1_	WT	12 L : 12 D	mRNA levels
*κ*_2_	WT	LL	period
*κ*_3_	WT	DD	period
*κ*_4_	*toc1*	LL	period
*κ*_5_	*ztl*	LL	period
*κ*_6_	*lhy*/*cca1*	DD	period
*κ*_7_	*prr7*/*prr9*	LL	period
*κ*_8_	*gi*	LL	period
*κ*_9_	*cop1*	LL	period

We now briefly outline how the constraints in part (i) are translated to our framework of constraints. We refer to the WT 12 L : 12 D GE-combination as *κ*_1_ (table 1). We let *g*_*κ*_1__(*t*, *k*) be the model solution representing the mRNA and protein levels of the system described by equation (2.1) with *κ* = *κ*_1_. The biological data for a particular mRNA represented by the *j*th variable in the model gives time-point measurements *T* = {*t*_1_, *t*_2_, *…* , *t_l_*} over a 24-h period. Each time-point measurement translates to a constraint *C_i_*(*k*) (*i* = 1, *…* , *l*) that represents the level of the model solution 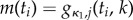 at a time-point *t_i_ ∈ T*. The equivalent linearized constraint for *i* = 1, *…* , *l* is
3.1
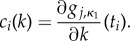



The mRNA profiles of nine genes measured at different time points of the light : dark cycle result in 82 linearized constraints of the type above. A detailed breakdown of the constraints coming from each gene measurement is left to the electronic supplementary material.

The reader might wonder why we do not use a constraint on the vector 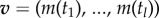 rather than what we do which is to regard these as individual constraints. The key point is that these measurements *m*(*t_i_*) will be highly correlated. We could assign a constraint value to the vector ***v*** but this would lose the information that some time points have much greater constraint value than others. This is confirmed in electronic supplementary material, table S2, because the constraint values of the individual constraints on the mRNA levels can vary by an order of magnitude or more. In this example and others, one can drop a majority of the time points with hardly any loss in accuracy.

The second type of constraints comes from values of free-run periods of the plants in different GE-backgrounds (cf. table 1). The constraint *C*(*k*) is the period *τ*(*k*) of the model solution 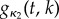 for a GE-background, *κ*_2_ (WT plant under LL). Thus, the linearization is *c*(*k*) = *∂τ*/*∂k*. This can be expressed in terms of the solution 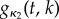 using any variable (e.g. *j*th variable) as follows from [7]. If 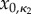 is a point on the limit cycle and the corresponding solution is given by 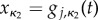 so that 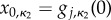 then
3.2
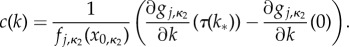



The Pokhilko model matched the period data for the clock in eight different GE-combinations [6]. A breakdown of period data fitted and reproduced without fitting is listed in table 1. Out of the eight models (each associated with one GE-combination), only four had long-term stable oscillations and thus only their period profiles can be translated to our framework to make up four linearized constraints. These are the period constraints of GE-combinations *κ*_2_, *κ*_4_, *κ*_5_ and *κ*_7_.

Several of the GE-combinations in table 1 describe a plant model with a mutant genetic background. To convert a WT model to a mutant model, the convention is to set the translation rate of the knocked-out gene, *k_j_*, to be either zero or sufficiently close to zero. While for a WT model, all parameters can be perturbed, in mutant models, we do not allow the translation rate of the knocked-out gene to be perturbed, as this rate essentially describes the mutant model. This means that for a constraint *c_i_* of a mutant model, we must set the corresponding (*j*th) entry to zero, i.e. *c_i_*_,*j*_(*k*) = 0.

When calculating these constraints, we noted that, before normalization, many had very small norms as is shown in figure 1. As a small norm means that the constraint only varies by a small amount when parameters are varied, such constraints are ineffective and therefore should not be used. We return to this in §4 where we explain why it is reasonable to expect that many constraints will be forced to have a small norm.

We scale the constraints by standard errors in the case of time-series measurements and by standard deviations or standard errors (depending on which is available in the literature) in the case of the period constraints. As the time series are normalized so that peak value is 1, the standard errors are normalized by the same factor as the time series. More details are given in the electronic supplementary material.

**Figure 2.**
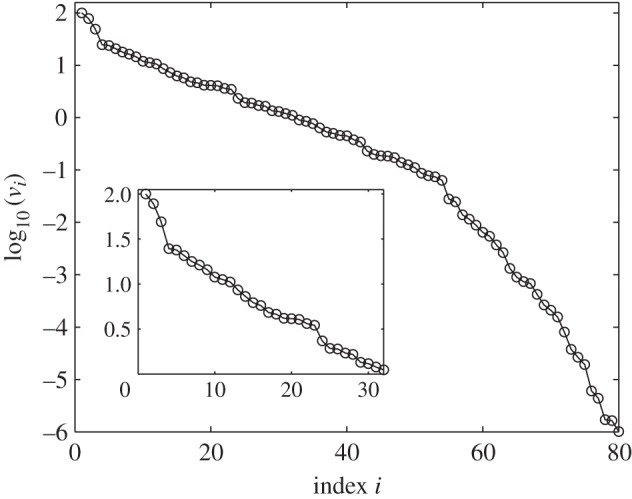
Values of the top 80 Pokhilko model constraints in order of decreasing value, 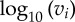 (open circles). Inset shows the top 32 ordered constraints.

In total, the Pokhilko model has 86 linearized constraints and the constraint values of the ranked linearized constraints are exponentially decreasing (figure 2). The ranking reveals that there is little value in using more than the top 32 constraints (inset in figure 2) as the remaining 54 constraints have constraint values of less than 1% of the top ranked constraint value.

The top four constraints are the constraints from GE-combinations *κ*_1_ relating to the levels of GI mRNA, and the period constraints of the model for GE-combinations *κ*_2_ and *κ*_5_ (described in table 1). The full list of the top 20 constraints is given in electronic supplementary material, table S2. All four period constraints of the model feature in the list of top 20 constraints.

Ranking also reveals that there is a large jump in the constraint values, with the six ranked lowest having near-zero constraint values. This set of six constraints comprises a combination of constraints on LUX and ELF4 mRNA levels of a model of GE-combination *κ*_1_ (WT plant entrained to 12 L : 12 D). Closer inspection of the constraints reveals that the linearized constraints on LUX mRNA levels are nearly identical to the constraints on ELF4 mRNA levels. This is not surprising, as the ODE equations describing these two mRNAs are almost identical. They share the same transcription term and kinetic constants of (linear) degradation rates and therefore these constraints are effectively identical.

**Figure 3.**
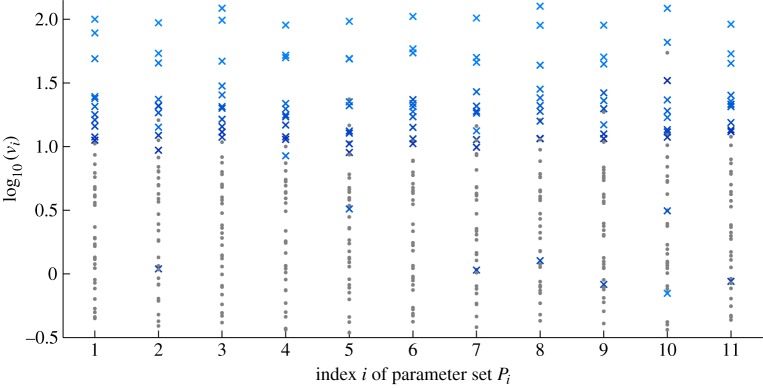
Values of the top 40 constraints of the Pokhilko 2012 model for different parameter sets. *P*_1_ set is the original model parameter set. The top 10 constraints are identified in blue crosses, with progressively darker shade of blue indicating the lower rank of the constraint. These same constraints are identified in models with different parameter sets (*P_i_*, *i* = 2, … , 11), based on perturbations of *P*_1_. The remaining 30 constraints are plotted as grey circles.

We also check how much the ranking of the constraints changes when we perturb the model parameters. Each new parameter set is obtained by perturbing every parameter *k_j_* from its original value (from [6]) by adding a perturbation which is normally distributed with mean zero and standard deviation 0.05 *k_j_* (further details are described in the electronic supplementary material). Under these parameter perturbations, the models appear to maintain a near-identical constraint ranking of the top 10 constraints to the ranking of the original model. Figure 3 shows the top 40 constraint values for 10 models simulated under different parameter sets *P_i_* chosen in this way with the top 10 constraints of the original model (*P*_1_) identified by crosses shaded in blue (with progressively darker shading indicating lower rank of the associated constraint). These same constraints were identified in the other 10 models (*P_i_*, *i* = 2, *…* , 11) and they appear to feature mainly among the top 10 constraints and to mainly preserve the rank order. It is also worth noting that aside from the very similar rank order, each of the 10 models preserved the exponential decay in the constraint values of the constraints. This result indicates that the rankings and the rate of decay of constraint values are robust to parameter perturbations.

### 3.2. Locke 2006 model of *Arabidopsis thaliana* circadian clock

The Locke 2006 model [8] is an earlier plant clock model that describes interaction of a subset of genes from the Pokhilko model and has *n* = 16 state variables. The model has *s* = 77 parameters, most of which correspond to various kinetic rates and all of which can be perturbed, as even the Hill coefficients are fitted (cf. Pokhilko model). It is interesting to consider this alongside the Pokhilko model because it is fitted to qualitative features of the data, for example the shape of the mRNA expressed through broadness of the troughs and sharpness of the peaks. Other features fitted include amplitude of oscillations, timing of peak and trough mRNA levels, and period of oscillations.

A typical constraint on oscillation period was given in the previous subsection (equation (3.2)), while a constraint on amplitude can easily be obtained from constraints on solution levels (equation (3.1)). To define broadness of peaks and troughs for a variable of interest, we followed the description in [8]. Locke *et al.* describe the difference in the value of a particular variable in *g_κ_*(*t*, *k*) 2 h before and after the peak value time. For a sharp peak, the expectation is for the variable levels to fall quickly on either side of the peak. Consider the *j*th variable of *g*_*κ*_(*t*, *k*) and define the ratio of how fast the level of *g*_*j*κ_(*t*, *k*) falls 2 h after time of its peak *ϕ_j_* by,

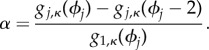



Together with the constraint on the timing of the peak,
3.3
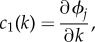

constraining
3.4
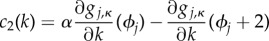

ensures that the ratio of levels of the *j*th variable at the two time points is fixed.

As we can calculate the partial derivative with respect to parameters of any variable of solution *g_j_*_,_*_*κ*_*(*t*, *k*) at any time point, we can derive similar constraints to fix the ratio of levels at any time. Note that the constraint of the peak timing (equation (3.3)) can also be obtained from the partial derivatives *∂g_j_*_,__*κ*_/*∂k*, [7] q.v. This mathematical description, as well as the full description of other constraints listed above are given in the electronic supplementary material.

The full list of GE-combinations and the constraints is given in the electronic supplementary material. Construction of mutant models and their constraints follows closely the description we outlined above for the Pokhilko model. In the mutant versions of the Locke model, whole sub-networks of the clock can become non-functional, i.e. multiple model variables converge to the zero equilibrium. This means that the relevant model structure can be reduced and the effect of fewer parameters needs to be considered in the constraints (i.e. more entries of the linearized constraints can be set to zero). The full list of these types of reductions for Locke mutant models is outlined in the electronic supplementary material.

The data presented in [8] does not have any error bars, so it is not possible to extract any error measurements pertaining to the shape of the oscillations and their peak times. We describe how we determined the s.e. in the electronic supplementary material.

**Figure 4.**
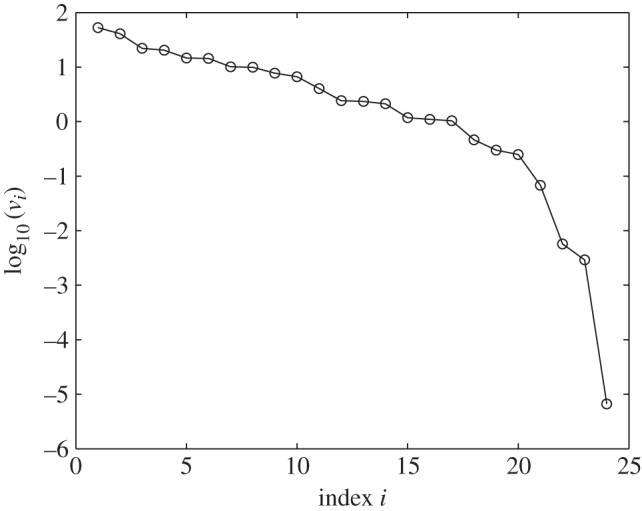
Values of the Locke model constraints, 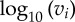, in the order of decreasing value (open circles).

The Locke model has 24 linearized constraints and their ranking according to constraint value also shows an exponential decrease (figure 4).

Only the top 17 constraints show any significant constraint value, with the value of the 17th constraint at 1.96% of the highest value. The top five constraints are associated with the period of the WT clock in LL, LHY/CCA1 amplitude in WT 12 L : 12 D, the period of the *lhy/cca1* mutant clock in DD, the level of LHY/CCA1 fall after peak in the *toc1* mutant clock in 12 L : 12 D and the period of the *toc1* mutant plant in DD. The full list of top constraints is presented in electronic supplementary material, table S4. It is worth noting that the period constraints for WT LL and both mutants, *lhy/cca1* and *toc1*, feature at the top of the rankings. It is not possible to compare the ranking of the constraints from the Locke and Pokhilko models because, even though they do model the same biological system, their constraints are very different. However, it is worth noting that the two constraints that feature in both models (periods of the WT and the *toc1* mutant clocks in LL conditions) are featured at the top of both rankings.

### 3.3. NF-*κ*B signalling system

We consider the model of the NF-*κ*B system from [9]. The solution of interest is a transient solution describing the oscillations in the level of cytoplasmic and nuclear NF-*κ*B concentration resulting from an incoming signal of tumour-necrosis factor-*α* (TNF*α*). The system is compared to experiments where cells were subjected to constant and pulsatile TNF*α* signals for total periods of approximately 600 min. Salient characteristics of the ratio of nuclear to cytoplasmic NF-*κ*B (henceforth denoted by N : C NF-*κ*B) were identified and the model constructed to match the observed characteristics.

N : C NF-*κ*B does not feature as a variable of the NF-*κ*B model, hence we introduce an additional ODE for the dynamics of N : C NF-*κ*B and consequently the number *n* of state variables is 16. Other state variables include those describing cytoplasmic and nuclear NF-*κ*B, I*κ*B*α*, their complexes, and also the A20 gene and the kinase IKK and its activated and inactivated states. The IKK system is activated downstream of the TNF*α* receptors and this, in turn, causes phosphorylation and subsequent degradation of I*κ*B freeing NF-*κ*B to enter the nucleus. This activates I*κ*B*α* transcription and the subsequent production of I*κ*B*α* protein that binds the nuclear NF-*κ*B and pulls it back into the cytoplasm, restarting the cycle.

The model has *s* = 28 parameters, most of which are rate constants. Parameter values were fitted to match the observed N : C NF-*κ*B oscillatory responses such as peak timing, persistence in oscillations and specific decay in oscillation amplitude. The various GE-combinations and the full list of associated constraints are shown in the electronic supplementary material.

The NF-*κ*B model of Ashall *et al*. [9] was fit using specific cost functions (described in electronic supplementary material, table S5 of [9]). A score of 1 is set to approximately match 1 s.d. from the mean of the respective feature (that they wish to match). From this information, we could extract the standard errors which we used to scale the model constraints. Further details are given in the electronic supplementary material.

**Figure 5.**
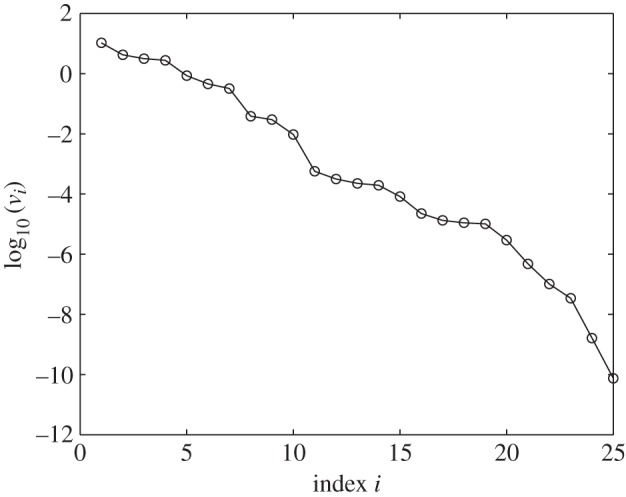
Values of the NF-*κ*B model constraints, 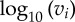, in the order of decreasing value (open circles).

The key observed characteristics of the model translate to 25 linearized constraints, details of which are given in the electronic supplementary material. Some of the target characteristics for model parameter fitting that are outlined in [9] could be eliminated (more information about that elimination is given in electronic supplementary material). The 25 linearized constraints are ranked in order of decreasing constraint values in figure 5 and they show an exponential decrease in constraint values. Only the top seven constraints (listed in table 2) have any significant constraint value (i.e. their own values are higher than 1% of the top value).

**Table 2.** Top 7 ranked constraints of the NF-*κ*B model of [9].

ranking	constraint	GE-combination
1	ratio of second-to-first peak level	100-min repeat pulse
2	ratio of second-to-first peak level	200-min repeat pulse
3	level of first peak	100-min repeat pulse
4	ratio of second-to-first peak level	60-min repeat pulse
5	ratio of third-to-first peak level	60-min repeat pulse
6	level of fifth peak	constant pulse
7	ratio of third-to-first peak level	100-min repeat pulse

## 4. Ordered constraints tend to have rapidly decreasing constraint values and many unnormalized constraints have a small norm

The examples of §3 manifest the two properties mentioned at the beginning of the paper, namely that the constraint values decrease rapidly and that many unnormalized constraints have a small norm. We now explain why this is the case.

It has been observed [7] that a large class of models of regulatory and signalling systems of the sort that we are considering have the following property: there is (i) a rapidly decreasing sequence of *s* positive numbers, 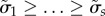, (ii) *s n*-dimensional time series defined for 0 ≤ *t* ≤ *T*, *U_i_*(*t*) = (*U_i_*_,1_(*t*), *…* , *U_i_*_,*n*_(*t*)), *i* = 1, *…* , *s*, which are of unit length and orthogonal to each other in the *L*_2_ sense, and (iii) an orthogonal *s* × *s* matrix *W*, such that for any change in parameters *k → k* + *δk*, the corresponding change *δg* in the solution of interest is
4.1
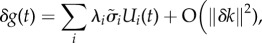

where 
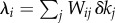
. This result comes from the singular value decomposition 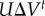 of the linearization of the map from parameters *k* to the solution of interest. The columns of *U* are the time series *U_i_*(*t*), *V* = *W^t^* is a *s* × *s* orthogonal matrix and Δ is a diagonal matrix with entries 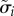. In [7], this observation is formulated for a model with a single GE-combination, however the same decomposition will apply for a model where multiple GE combinations are integrated, though here the decay of the singular values 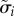 may be slower. The relevant result in [7] is expressed as in (4.1). However, such a result is implicitly contained in the earlier papers [10,11] because the 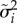 are eigenvalues of the Fisher information matrix (FIM) discussed there. They showed that they decrease quickly in some systems biology models. This observation was developed further in [12–14]. The decay was also found early on in the context of circadian clocks in [15,16].

It is shown in the electronic supplementary material that for such a model under very general conditions, an ordered set of constraints *c*_1_, *…* , *c_m_* will have

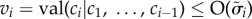

and thus that the rapid decline of the 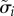 implies rapid decline of the constraint values *v_i_*. This assumes that the constraints are functions of the parameters through their dependence on the solution of interest of equation (2.1) or (2.2).

As the reader will see from the figures, there is no natural gap in the constraint values *v_i_*. However, there is a natural cut-off given by 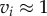 because if 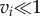, the unconstrained variation defined by the constraint value is small compared with the uncertainty in the constraint.

We mentioned above that many constraints that have been used to analyse the systems above have linearizations with a very small norm. When we observed this, we realized that one can argue that this is a consequence of equation (4.1). As the constraints *C_i_* are functions of the solution of interest *g*, i.e. 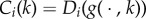, it follows from (4.1) that the linearized constraints satisfy 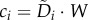, where 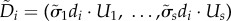 and 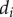 is the derivative of 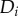 with respect to *g* evaluated at 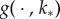 (see section 3 of the electronic supplementary material). Therefore, as *W* is orthonormal,
4.2
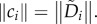



As the *U_i_* are orthogonal, it seems reasonable to assume that 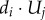 is uncorrelated with 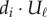 if 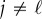. If we assume that the norms of the 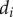 are O(1), then we can model the 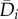 as random *s*-dimensional vectors with O(1) norm. As is explained in the electronic supplementary material, it follows from this that, with high probability not less than 1 − O(e*^−*ɛ*s^*^/4^),

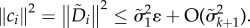



In the electronic supplementary material, this is illustrated with an example showing the expected distribution of norms 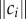 under these assumptions.

As constraints are not very useful, if their norm is small compared with their uncertainty, we already get some very useful information by just checking these norms. Indeed, we see that about 75 of the constraints on the Pokhilko 2012 model have norms less than 10% of the norm of the constraint with the greatest norm. For the Locke and NF-κB models, about 50% of the constraints are this small.

## 5. The geometric interpretation of constraint value

### 5.1. Geometric shape of the approximate solution set

We provide a geometric interpretation of constraint value when *m* ≤ *s* by considering the geometric shape of the approximate solution set. When *m* > *s*, this set might be empty. Consider the mapping 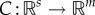 given by *C*(*k*) = (*C*_1_(*k*), *…* , *C_m_*(*k*)). We assume that the corresponding linear constraints *c*_1_, *…* , *c_m_* are ordered and that the matrix *M* = *M*(*c*_1_, *…* ,*c_m_*) has maximal rank. Let *σ*_1_ ≥ *σ*_2_ ≥ … ≥ *σ_m_* > 0 be its positive singular values and let *V_i_* and *U_i_* be its right and left singular vectors.

In this case, as *M* is of maximal rank, the set Σ of parameters values which satisfy the constraints will, near the parameter vector of interest *k*_∗_ be a (*s* − *m*)-dimensional sub-manifold of the parameter space.

In the electronic supplementary material, theorem S4, we prove that the set of parameter values that approximately satisfy the constraints,

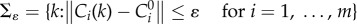

tends in a precise sense as *ɛ →* 0 to the set 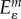 given by
5.1
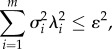

where *λ* and the parameters *k* are related by the equation *λ* = *W ·* (*k* − *k*_∗_), i.e. 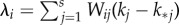, where *W* is an orthogonal matrix. This orthogonality is important because it ensures that objects in the *λ* coordinate system are measured on the same scale as in the original coordinate system. Therefore, *E_ɛ_* is the interior of an *m*-dimensional ellipsoid with principal axes of length *σ_i_* in both coordinate systems.

Therefore, we can interpret the effectiveness of the constraints as follows. The constraints only constrain the parameter values insofar as they constrain the *λ_i_* and the extent of this is that (i) *λ*_1_, *…* , *λ_m_* must satisfy equation (5.1) (i.e. that (*λ*_1_, *…* , *λ_m_*) regarded as a point in 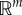 must be inside the ellipsoid 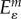 given by equation (5.1)) and (ii) *λ*_*m*+1_, *…* , *λ_s_* are unconstrained.

For an ordered set of linear constraints the notion of constraint value fits nicely with this interpretation because our *m → m* + 1 Transition theorem tells us that adding a constraint *C_m_*_+__1_ with linearization *c_m_*_+__1_ and constraint value

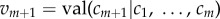

changes all the singular values by at most a O(1) scaling and adds a new singular value which is of the order *σ* = *v_m_*_+__1_. Thus, it adds a new principal axis to Σ*_*ɛ*_* the size of which is of the order of 1/*v_m_*_+1_.

## 6. Construction of optimization functions

We consider functions of the form
6.1
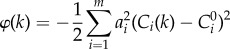

and suppose that *k*_∗_ is a maximum of this function. If *m* ≥ *s*, and the matrix *M* = *M*(*c*_1_, … , *c_m_*) has full rank *s*, then the structure of *φ* about its minimum is given by its Hessian. The Hessian is the matrix *F* of partial derivatives (*∂*^2^*ϕ*/*∂k_i_∂k_j_*) evaluated at *k*_∗_.

Without any loss of generality, we can incorporate the coefficients *a_i_* into the constraints and thereby assume that *a_i_* = 1. The second derivatives of *φ* are given by
6.2
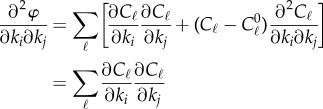

when *k* = *k*_∗_. If *c*_1_, … , *c_m_* are the linearized constraints associated with *C*_1_, … , *C_m_* at *k*_∗_ and *M* = *M*(*c*_1_, … , *c_m_*), then the right-hand term of (6.2) is the *ij*th entry of the matrix *F* = *M*^t^*M*, where *M*^t^ is the transpose of *M*. Thus, we see that if *m* < *s*, then *F* has zero eigenvalues and the Hessian of *φ* is degenerate. Thus, we now consider the case *m* ≥ *s* but mention the alternative case in a note below. Indeed, it is worth noting that in some applications (e.g. in [4,5]), the functions *φ* used are of the form in (6.1) with *m* < *s*.

Alternatively, one can use the function *ϕ* as an artificial likelihood and regard 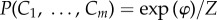 as the (normal) distribution of the vectors *C* = (*C*_1_, … , *C_m_*) (*Z* is the normalizing factor). In this case, the matrix *F* is the FIM for the system, i.e. *F* is the *P*-expectation of the Hessian of *−V* = −log*P* which is given by

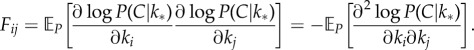



The inverse of the FIM provides an approximation of the covariance (i.e. the multidimensional spread around the mode) of the posterior probability distribution 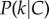 and provides a lower bound (generally known as the Cramér-Rao bound) for the error covariance of any unbiased estimator of the true parameters.

In either case, we are interested in the singular values 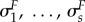 and singular vectors 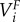 of *F* = M^*^M. However, the singular values of *F* are just the squares of the singular values for *M* and the singular vectors 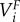 are the right singular vectors of *M*. The singular values and singular vectors of *F* characterize the nature of *φ* near its maximum. For example, close to the maximum, the level hypersurfaces of *φ* are approximated by the hypersurfaces
6.3
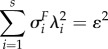

where 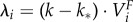. This tells us that *k* is well constrained in the directions 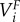 with 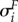 large and badly constrained in the directions 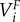, where 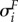 is small. This approximation result follows from well-known results about so-called Morse functions and arguments similar to those used in electronic supplementary material, theorem S4.

In particular, we are interested in how the singular values change when we remove or add a new constraint to *φ*. To determine whether to add a new constraint in the case of *m* ≥ *s*, one should firstly reorder the constraints using the algorithm defined by electronic supplementary material theorem S2, but using the constraints *c*_1_, … , *c_m_*_+1_ instead of *c*_1_, … , *c_m_*. This is because, when reordered, the new constraint may move much higher up the list and have a greater constraint value. One can then use the reordered list of constraints 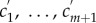 after possibly deleting those with the lowest values 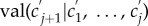.

Although above we are restricted to the case *m* ≥ *s*, the discussion above does apply to the case *m* < *s* if one restricts parameter changes that are allowed to be only those that do not change the linear combinations *λ_m_*_+1_, … , *λ_s_* of the parameters.

In this case, we use the *m → m* + 1 Transition theorem (electronic supplementary material, theorem S5) to address how the singular values change when we remove or add a new constraint to *φ*. The Transition theorem tells us that adding a new constraint *C_m_*_+1_ with linearization *c_m_*_+1_ and constraint value 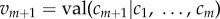 has the following effect. Consider the singular values 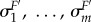 of the new matrix *F’*, where 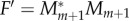 with *M_m_*_+1_ = *M*(*c*_1_, … , *c_m_*, *c_m_*_+1_). These have an interlacing property in that

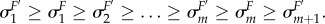

and moreover, in the electronic supplementary material, we show that 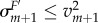 while for 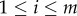


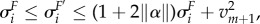

where *α* = (*α*_1_, … *α_m_*) is such that 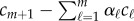 is normal to *c*_1_, … , *c_m_*.

A simple way to characterize the effectiveness of *φ* is via the condition number of the matrix *F* which can be taken to be given by 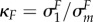 as this determines the ratio of the lengths of the major and minor axes of the ellipsoid given by (6.3). Using the above inequalities, we see that

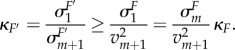



Therefore, to improve the condition number, one must find new constraints whose constraint value exceeds the smallest singular value of *M*.

This quantifies the usefulness of adding a new constraint. It is only useful when its reordered constraint value is relatively high. Using a low-value constraint involves extra computational cost with no significant improvement in terms of estimation utility. Moreover, because of the results of §4 finding constraints with good reordered constraint value will require careful design.

## 7. Experimental optimization

Experimental design in systems biology has been discussed extensively from a number of points of view including classical approaches using Fisher information [17], sensitivity analysis [18] and methods to maximize the expected mutual information between prior and posterior parameter distributions ([19] and references therein). In this section, we illustrate how the constraint value can be used for experimental design. The idea is that once a working model has been formulated and the current constraints *C*_1_, … , *C_m_* analysed, then one can test new GE-combinations for new constraints *C* with a high value 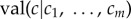. To do this, we formulate the model in equation (2.2) for all the relevant GE-combinations including the new one.

We now give some illustrative examples. In each case a gene mutant is simulated by putting the corresponding translation rate to zero and not allowing this rate to change when perturbing the parameters.

### 7.1. Pokhilko 2012 model and the *prr9* and *ni* mutants

No constraints for these mutants were used in formulating the Pokhilko 2012 model. One can therefore ask whether an experiment on the mutants will add value. When this experiment is being considered, we could predict the value of it for our purposes using our techniques and this can be used to help assess the priority of this experiment. In fact, the periods of these mutants are already known [20], but the discussion still illustrates our approach had we not had the data already. Moreover, given that we have it, we can also ask whether if it does add value and whether one should put in the effort to reparametrize the model to match it.

Therefore, using the Pokhilko 2012 model, we simulated the *prr9* mutant and *ni* mutant models in constant light. In fact, Salomé & McClung [20] have measured periods of the both *prr9* and *prr5* mutants (*prr5* is a proxy for our *ni* component) in three different clock markers. They estimate the period of the *prr9* mutant to range from 25.3 h ± 0.1 s.e. to 26.2 h ± 0.4 s.e. for the different markers. The model *prr9* mutant period is 23.90 h, slightly shorter than the estimated periods. The period constraint for the *prr9* mutant is calculated as explained above and it is scaled by the larger s.e. (i.e. 0.4). When compared to the other model constraints, the scaled *prr9* period constraint has very high constraint value. By order of decreasing value, it is the fourth highest constraint, with the constraint value approximately 26% of the top constraint value.

Salomé & McClung [20] also estimate the period of the *prr5* mutant to range from 23.1 h ± 0.1 s.e. to 23.9 h ± 0.2 s.e. for the different markers. In the model, the NI component is meant to be the proxy for PRR5. The model *ni* mutant period is 24.3281 h and so, within the estimated ranges. The period constraint for the *ni* mutant is calculated as explained above and it is scaled by the larger s.e. (i.e. 0.2). When compared to the other model constraints, the scaled *ni* period constraint has the highest constraint value and ranks as the most influential constraint. We thus conclude that, from this point of view, both the knockout experiments have significant value.

### 7.2. A20 knockdown for NF-*κ*B

A similar approach can be used with the NF-*κ*B model. As an example, we test whether the predictive constraint on the period of the A20-knockout-mutant model under constant TNF*α* adds value compared to the other constraints. The A20 mutant model is simulated by halving the transcription of A20. While the WT model under constant TNF*α* has a period of 93.95 min (calculated as the average peak distance from third to last (in this case, sixth) peak), the A20 mutant has a shorter period of 85.07 min (calculated as the average peak distance from third to last (in this case, seventh) peak). The period constraint is calculated and the entry relevant to A20 translation in the constraint is set to 0 (as this rate is not allowed to change in the mutant). The A20 predictive constraint ranks as the 12th top constraint (in order of decreasing constraint value). Its value is a lot lower than that of the top constraint value, approximately 0.0031% of the top value. Our model prediction is that this constraint does not add much value to the models given that the other constraints have been applied.

## 8. Discussion

There is a huge literature on fitting ODE systems to data and the relevant literature is simply too extensive to list. A key reference is [21] which initiated one of the main lines of enquiry in this area and [22] is a recent example of this with a good reference list. Methods using stochastic simulation such as MCMC, a Bayesian approach and/or hierarchical models have also been increasingly used, and [23,24] are examples of this. These methods generally employ a single likelihood or likelihood-like objective function as opposed to our approach which considers the optimization problem in terms of a set of many individual constraints. Moreover, they are so far only applied to relatively small systems. Similar questions are also being actively pursued for fully fledged stochastic models and this is currently a very active area of research [25–31]. A link between these two approaches and a possible way to move to bigger systems is given by the ABC methods and the ideas in this paper may aid the move to larger systems by helping construct good likelihoods and enabling better understanding of the shape of likelihood and optimization functions.

The examples that we discuss show that our approach gives a substantial amount of valuable information on the value of constraints, information that is very difficult, if not impossible, to obtain by intuition. They show that for these large state-of-the-art models, only a fraction of the constraints have non-negligible constraint values and they identify which of the constraints are valuable. This knowledge is extremely useful when fitting models and allows for a more rational approach. The examples given also demonstrate that this approach can be successfully applied to both quantitative and qualitative constraints.

We have demonstrated the non-intuitive fact that one should expect the constraint value of many constraints to be small and consequently ineffective. We characterized what can be learned from this approach in terms of understanding the geometry of the optimization problem, design of optimization functions and artificial likelihoods and experimental optimization. One can also use this theory to give useful information on how to optimize a non-optimal system using both deterministic and stochastic approaches. For example, when using deterministic gradient following methods, it is well-known [32] that a common problem is that the algorithms of the successive line minimization type are ineffective when the level surfaces of the constraints or optimization function have a ellipsoidal structure with an extreme aspect ratio of the sort we find. Our results suggest methods for choosing the move direction. Moreover, the most effective methods for moving to an optimum use Newton's method and this relies on inverting the derivative of the constraint map. As this is our matrix *M*(*c*_1_, … , *c_m_*), its smallest singular value tells us how well controlled the Newton algorithm will be. Finally, statistical optimization methods can use an artificial likelihood of the type we have analysed.

The fact that in a typical model, only a few constraints will have a significant value leads to an interesting new concept of a *tight model*. Suppose that we have an ordered set of constraints *C*_1_, … , *C_m_* and that *C_r_*_+1_, … , *C_m_* have very small constraint values. Furthermore, suppose that we have tuned the parameters so that *C*_1_, … , *C_r_* are satisfied. If we then demand that any further parameter changes must not change *C*_1_, … , *C_r_*, it will be extremely difficult to tune *C_r_*_+1_, … , *C_m_* because of their very small constraint values. Therefore, if *C_r_*_+1_, … , *C_m_* are quantitatively correct, this can be interpreted as suggesting that the structure of the model is correct. If the correctness of these small value constraints has not been artificially determined, then it is reasonable to define such a model as tight in the sense that a large number of constraints take the correct value even though only a proportion of them can be tuned by adjusting parameters.

If system biologists are to reliably use complex models to provide robust understanding, it is crucial that there are analytical tools to enable a rigorous assessment of the quality and selection of these models and their fit to current biological knowledge and data. Our aim in this paper is to contribute to that.

## Supplementary Material

Supplementary Information for Using constraints and their value for optimisation of large ode systems
