# Supplementary material for: Using constraints and their value for optimization of large ODE systems
Source: J R Soc Interface. 2015 Mar 6;12(104):20141303. doi: 10.1098/rsif.2014.1303 (PMC4345496; doi:10.1098/rsif.2014.1303)
Supplement: Supplementary Information for Using constraints and their value for optimisation of large ode systems [file rsif20141303supp1.pdf]

Supplementary Information for Using constraints and their value for optimisation of large ODE systems

Mirela Domijan [*] [†] **and David A. Rand** [*] [‡]

[*]Warwick Systems Biology Centre, University of Warwick, Coventry CV4 7AL, United Kingdom,[‡]Mathematics Institute, University of Warwick, Coventry CV4 7AL, United Kingdom, and [†]current address: The Sainsbury Laboratory, University of Cambridge, Cambridge CB2 1NN, United Kingdom

1. Singular Value Decomposition (SVD)

Singular Value Decomposition (SVD) gives a decomposition of the $m \times s$ matrix M into a product of the form $M = UDV^t$ where U is a $m \times s$ column-orthonormal matrix ($U^tU = I_s$), V is a $s \times s$ orthonormal matrix and $D = \operatorname{diag}(\sigma_1, \ldots, \sigma_s)$ is a diagonal matrix. This is the version of SVD that is often called thin SVD (see [18] for more details). The elements $\sigma_1 \geq \cdots \geq \sigma_s$ are the *singular values* of M. We note that the columns $V(:, j)$, $j = 1, \ldots s$ of V are an orthonormal basis for the parameter space $\mathbb{R}^s$. If the last k of the singular values are zero then the last k columns are an orthonormal basis for the kernel of M. If $m < s$ then $k \geq s - m$.

In what follows we will refer to the column vectors $V_j = V(:, j)$ and $U_j = U(:, j)$ as the right and left singular vectors respectively.

Notation. For such a matrix M we denote the ith singular value by $\sigma_i(M)$.

A result we will use a number of times is the following so-called interlacing property for singular values.

Interlacing property for singular values. Let A be any $m \times n$ matrix and A_r be a submatrix of A obtained by deleting r rows and/or columns. Then

$$\sigma_i(A) \leq \sigma_i(A_r) \leq \sigma_{i+r}(A), \quad i = 1, \ldots \min\{m, n\}. \qquad [1]$$

See Corollary 3.1.3 of [8].

We denote by sdet$M$ the product of the non-zero singular values of M. For a square non-singular matrix sdet$M$ is the the magnitude of the determinant.

Theorem 1. *Suppose that M is any $m \times s$ matrix ($m < s$) with rows $r_1, \ldots, r_m$. If and M' is M with an extra row r_{m+1} added, then, under the condition that r_{m+1} is linearly independent of $r_1, \ldots, r_m$,*

$$\frac{\mathrm{sdet}M'}{\mathrm{sdet}M} = ||n||$$

where n is the unique vector normal to the vectors $r_1, \ldots, r_m$ such that $r_{m+1} - n$ lies in the span of $r_1, \ldots, r_m$.

If M and M' are as above then we denote $||n||$ by $r(M'|M)$.

Proof. Let $M' = L'Q$ be the LQ-decomposition of M' so that L' is a lower-triangular matrix with non-negative diagonal entries and Q is orthogonal. Then $M = LQ$ where L is L' with the last row deleted. Since the product of the non-zero singular values of a lower-triangular matrix equals the product of its non-zero diagonal elements and since L has one extra diagonal element $L'_{m+1\,m+1}$,

$$\mathrm{sdet}M'/\mathrm{sdet}M = L'_{m+1\,m+1}.$$

But, if $L'_i = (L'_{ij})$ and Q_i are the ith rows of L' and Q, respectively,

$$r_{m+1} = L'_{m+1}.Q = \sum_{i=1}^{m+1} L'_{m+1\,i} Q_i.$$

Therefore, since $\sum_{i=1}^{m} L'_{m+1\,i} Q_i$ is in the span of $r_1, \ldots, r_m$, $n = L'_{m+1\,m+1} Q_{m+1}$ and $||n|| = L'_{m+1\,m+1}$. QED

2. Ordering constraints

Suppose that A is a $m \times s$. If $m \leq s$ then A is said to be upper triangular if all entries below the diagonal are zero. We generalise this a little to the case where $m > s$ by saying that A is upper triangular if it is of the form $[A_1 A_2]$ where A_1 is $s \times s$ and is upper triangular and A_2 is $(m - s) \times s$. A lower triangular matrix is one whose transpose is upper triangular.

As is well-known (e.g. [18]) LQ-decomposition with row pivoting produces a decomposition of any $m \times s$ matrix M as $M = PLQ$ where P is a permutation matrix, Q is a $s \times s$ orthogonal matrix and L is a lower-triangular $m \times s$ matrix with non-increasing diagonal elements $L_{11} \geq \cdots \geq L_{mm} \geq 0$. If the row rank of M is q then the first q of these are positive.

Theorem 2. *Any set of linearised constraints can be indexed, say as $c_1, \ldots, c_m$, so that the values $v_i = val(c_i|c_1, \ldots, c_{i-1})$ are decreasing i.e. $v_1 \geq v_2 \geq \ldots \geq v_m$. Then $v_i = |a_{ii}|$ where a_{ii} is the ith diagonal element of the LQ-decomposition of $M = M(c_1, \ldots, c_m)$ i.e. $M = AV$ where A is a $m \times s$ lower triangular matrix and V is a $s \times s$ orthogonal matrix. If V_i is the ith row of V, the vector $a_{ii}V_i$ equals $r(c_i|c_1, \ldots, c_{i-1})$. If $\sigma_1 \geq \ldots \geq \sigma_q$ are the non-zero singular values of M then $\prod_{i=1}^{q} \sigma_i = \prod_{i=1}^{q} v_i$.*

Proof. Now suppose we have a set of m constraints and that M is the matrix with these as rows. Let $M = PLQ$ be the LQ-decomposition of M. Let c_i be the ith row of P^tM, Then $c_1, \ldots, c_m$ are the same set of constraints but now are ordered. This is because

$$r(c_j|c_1, \ldots, c_{j-1}) = L_{jj}Q_j$$

where Q_j is the jth row of Q, and moreover,

$$v_j = \mathrm{val}(c_j|c_1, \ldots, c_{j-1}) = L_{jj}.$$

Finally we note that M and L have the same singular values since Q is orthonormal. Suppose that q of the L_{ii} are non-zero. Then the $q \times q$ matrix L' which is the top lefthand corner of L is lower-triangular and non-singular and its determinant is $L_{11} \cdots L_{qq}$. But this is the product of the singular values of L' and since these are precisely the non-zero singular values of L we deduce that sdet$M = L_{11} \cdots L_{qq}$ and that

$$\prod_{i=1}^{q} \sigma_i = \prod_{i=1}^{q} v_i. \qquad [2]$$

QED

3. Rapid decrease of value

Using the relation

$$\delta g(t)=\sum_{i=1}^{s}\lambda_i\tilde{\sigma}_i U_i(t)+O(||\delta k||^2) \qquad [3]$$

as described in the main text where $\lambda_i=\sum_j W_{ij}\,\delta k_j$, we show that

$$\mathrm{val}(c_{m+1}|c_1,\ldots,c_m)\le O(\tilde{\sigma}_{m+1}). \qquad [4]$$

In fact, we show that if there is a total of r constraints, $r\le s$, then $\mathrm{val}(c_{m+1}|c_1,\ldots,c_m)\le\kappa\tilde{\sigma}_{m+1}$ where κ is the norm of the matrix A_r defined below. Only the case $m\le s$ is relevant here as at most s constraint values are non-zero.

It follows from (3) and the assumption that $C_i(k)=D_i(g(\cdot,k))$ that the linearised constraints $c_i=(c_{i1},\cdots,c_{is})$ are of the form $c_{ij}=\sum_{l=1}^{s}\tilde{\sigma}_l W_{lj}\Delta_{il}$ where $\Delta_{il}=d_i\cdot U_l$ and d_i is the derivative of D_i with respect to g evaluated at $g(\cdot,k_*)$. In matrix form, $c_i=\tilde{D}_iW$ where $\tilde{D}_i=(\tilde{\sigma}_1 d_i\cdot U_1,\ldots,\tilde{\sigma}_s d_i\cdot U_s)$.

Therefore, for $m\le s$, the matrix $M_m=M(c_1,\ldots,c_m)$ is given by

$$M_m=\begin{pmatrix}\tilde{\sigma}_1 d_1\cdot U_1 & \cdots & \tilde{\sigma}_s d_1\cdot U_s\\ \vdots & \ddots & \vdots\\ \tilde{\sigma}_1 d_m\cdot U_1 & \cdots & \tilde{\sigma}_s d_m\cdot U_s\end{pmatrix}\times W \qquad [5]$$

$$=\begin{pmatrix}d_1\cdot U_1 & \cdots & d_1\cdot U_s\\ \vdots & \ddots & \vdots\\ d_m\cdot U_1 & \cdots & d_m\cdot U_s\end{pmatrix}\times\begin{pmatrix}\tilde{\sigma}_1 & \cdots & 0\\ \vdots & \ddots & \vdots\\ 0 & \cdots & \tilde{\sigma}_s\end{pmatrix}\times W$$

$$=A_m\tilde{\Sigma}W. \qquad [6]$$

Similarly, $M_{m+1}=A_{m+1}\tilde{\Sigma}W$.

Theorem 3. *In this case,*

$$v_{m+1}=\mathrm{val}(c_{m+1}|c_1,\ldots,c_m)\le\kappa\tilde{\sigma}_{m+1}$$

where $\kappa=||A_r||$.

Now let $\tilde{A}_m$ denote the $s\times s$ matrix given by adding $s-m$ zero rows below A_m. Then, $\sigma_i(\tilde{A}_m)=\sigma_i(A_m)$ and $\sigma_i(A_m\tilde{\Sigma})=\sigma_i(\tilde{A}_m\tilde{\Sigma})$ if $i\le m$. Since, the singular values of a matrix and its transpose are identical, $\sigma_i(\tilde{A}_m\tilde{\Sigma})=\sigma_i(\tilde{\Sigma}\tilde{A}_m^t)$. Thus, using Part (d) of Theorem 3.3.16 of [8] we have

$$\begin{aligned}\sigma_i(A_m\tilde{\Sigma})&=\sigma_i(\tilde{\Sigma}\tilde{A}_m^t)\le\sigma_i(\tilde{\Sigma})\sigma_1(\tilde{A}_m^t)\\ &=\tilde{\sigma}_i\sigma_1(A_m)\le\tilde{\sigma}_i\sigma_1(A_r)\end{aligned} \qquad [7]$$

where the last inequality follows from the interlacing property (1). The result follows since $\sigma_1(A_r)=||A_r||$ and $A_m\tilde{\Sigma}=M_mW^t$ has same singular values as M_m since W is orthogonal. QED

Note. One could have used the QL-decomposition of A_r to prove this since if $A_r=QL$, $M_r=QL\tilde{\Sigma}$ and $L\tilde{\Sigma}$ is lower triangular with diagonal entries $R_{ii}\tilde{\sigma}_i$ so that $\mathrm{sdet}M_r=R_{11}\cdots R_{rr}\tilde{\sigma}_1\cdots\tilde{\sigma}_r$.

4. Geometry of value and proof of Theorem 4

Recall that we consider the mapping $C:\mathbb{R}^s\to\mathbb{R}^m$ given by $C(k)=(C_1(k),\ldots,C_m(k))$. We assume that the corresponding linear constraints $c_1,\ldots,c_m$ are ordered and that the matrix $M=M(c_1,\ldots,c_m)$ has full rank and we let $M=UDV^t$ be its Singular Value Decomposition where U is a $m\times s$ column-orthonormal matrix ($U^tU=I_s$), V is a $s\times s$ orthonormal matrix and $D=\mathrm{diag}(\sigma_1,\ldots,\sigma_s)$ is a diagonal matrix with diagonal entries $\sigma_1\ge\sigma_2\ge\cdots\ge\sigma_m>0$. Let W be the transpose of V and $\lambda_1,\ldots\lambda_s$ be the new coordinate system about k_* given by $\lambda_i=\sum_{j=1}^{s}W_{ij}(k_j-k_{*j})$.

Theorem 4. *The set*

$$\Sigma_\varepsilon=\{k:\|C_i(k)-C_i^0\|\le\varepsilon\ \textit{for}\ i=1,\ldots,m\}$$

tends as $\varepsilon\to 0$ *to the set* E_ε^m *given by*

$$\sum_{i=1}^{m}\sigma_i^2\lambda_i^2\le\varepsilon^2 \qquad [8]$$

in the sense that

$$\lim_{\varepsilon\to 0}\frac{1}{\varepsilon}\Sigma_\varepsilon=E_1^m \qquad [9]$$

in the Hausdorff topology.

Before proceeding to the proof of this theorem we firstly prove a useful lemma. In the following S_ε^m denotes the m-dimensional ball $||x||\le\varepsilon$.

Lemma 1.

$$M^{-1}(S_\varepsilon^j)=\{\lambda:||D\cdot\lambda||\le\varepsilon\}.$$

Proof of Lemma 1. If $S=DV^t$,

$$\begin{aligned}||M\cdot\delta k||^2&=(M\cdot\delta k)^t(M\cdot\delta k)\\ &=(UDV^t\delta k)^t(UDV^t\delta k)\\ &=(DV^t\delta k)^t(DV^t\delta k)\\ &=||S\cdot\delta k||^2\end{aligned} \qquad [10]$$

because U is column-orthogonal. Thus $M^{-1}(S_\varepsilon^j)$ is given by $||S\cdot\delta k||\le\varepsilon$ and the lemma follows directly from this. QED

Proof of Theorem 4. Consider the diagram shown below. Let K be the kernel of M. The columns $V_1,\ldots,V_s$ of V provide an orthogonal basis of $\mathbb{R}^s$ such that $V_{m+1},\ldots,V_s$ span K. The columns of U provide an orthogonal basis $U_1,\ldots,U_m$ of $\mathbb{R}^m$ such that $M\cdot V_i=\sigma_iU_i$.

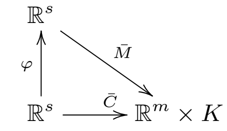

Define the maps $\bar{C}$ and $\bar{M}$ from $\mathbb{R}^s$ to $\mathbb{R}^m\times K$ by $\bar{C}(\delta k)=(C(\delta k+k_*)-C^0,\pi_K(\delta k))$ and $\bar{M}(\delta k)=(M\cdot\delta k,\pi_K(\delta k))$ where π_K is the orthogonal projection $\pi_K:\mathbb{R}^s\to K$ and $\delta k=k-k_*$. Then since M is the derivative of C at k_* and has full rank, (i) $\bar{C}$ and $\bar{M}$ are local diffeomorphisms at k_*, and (ii) $\varphi=\bar{M}^{-1}\circ\bar{C}$ is a local diffeomorphism whose derivative at k_* is the identity.

We are interested in

$$\Sigma_\varepsilon=\{k:\|C_i(k)-C_i^0\|\le\varepsilon\ \text{for}\ i=1,\ldots,m\}$$

which is $C^{-1}(C^0+S_\varepsilon^m)$ where $C^0=(C_1^0,\ldots,C_m^0)$ and S_ε^m is the set $||x||<\varepsilon$ in $\mathbb{R}^m$. This equals

$$\bar{C}^{-1}(S_\varepsilon^m\times K)=\varphi^{-1}(\bar{M}^{-1}(S_\varepsilon^m\times K))=\varphi^{-1}(M^{-1}(S_\varepsilon^m)).$$

But by Lemma 1, $M^{-1}(S_\varepsilon^m)$ is given by

$$\sum_{i=1}^{m}\sigma_i^2\lambda_i^2\le\varepsilon^2 \qquad [11]$$

Let $\psi_\varepsilon(x)=\varepsilon^{-1}\varphi(\varepsilon x)$. Since $d\varphi(k_*)$ is the identity and φ is C^2, ψ_ε converges to the identity in the Hölder space $C^{1+\alpha}$ on the ball $||x||\le 2$. But $\varepsilon^{-1}\varphi^{-1}(E_\varepsilon^m)=\psi_\varepsilon^{-1}(S_1^m)$ and therefore $\lim_{\varepsilon\to 0}\varepsilon^{-1}\Sigma_\varepsilon$ is given by $\sum_{i=1}^{m}\sigma_i^2\lambda_i^2\le 1$. QED

Constraining parameters. The above theorem enables us to understand how the parameters are constrained by the conditions $\|C_i(k) - C_i^0\| \leq \varepsilon$ for $i = 1, \ldots, m$. The theorem tells us that the set of such k is approximately given by equation (11) where $\lambda = V^t \cdot (k - k_*)$. It follows that these conditions do not constrain $\lambda_{m+1}, \ldots, \lambda_s$. Let E_ε^m be the subset of Σ_ε given by $\lambda_{m+1} = \cdots = \lambda_s = 0$. This is an ellipsoid in $\mathbb{R}^m$ and its shape characterises the information coming from the constraints $c_1, \ldots, c_m$. But E_ε^m is characterised by the directions and lengths of its semi-principal axes and these are given by the singular values of M.

The volume, in n-dimensional Euclidean space, of the unit n-ball, D_n is given by

$$\mathrm{Vol}(D_n) = \frac{\pi^{n/2}}{\Gamma(\frac{n}{2}+1)}$$

where Γ is the gamma function. The volume of the n-ball of radius ε is therefore $\varepsilon^n \mathrm{Vol}(D_n)$ and therefore the volume of the ellipsoid E_ε^m is

$$\mathrm{Vol}(E_\varepsilon^m) = \mathrm{Vol}(S_\varepsilon^m)\prod_{i=1}^m \sigma_i = \mathrm{Vol}(S_\varepsilon^m)\prod_{i=1}^m v_i.$$

The second equality follows from Equation (2).

5. Constraint values and singular values

We consider what happens when we add the new constraint c_{m+1} to an ordered set of constraints $c_1, \ldots, c_m$. This set of constraints may be degenerate in which case we will have $\mathrm{val}(c_{r+1}|c_1, \ldots, c_r) = 0$ for some $r \leq m$. It follows that

$$c_{m+1} = \sum_{\ell=1}^m \alpha_\ell c_\ell + n$$

where $n \neq 0$ is normal to the c_i for $i \leq m$ or $n = 0$, and that $v_{m+1} = ||n|| = \mathrm{val}(c_{m+1}|c_1, \ldots, c_m)$. The following theorem, tells us how the value of the new constraint corresponds to the singular values of $M_m = M(c_1, \ldots, c_m)$ and $M_{m+1} = M(c_1, \ldots, c_m, c_{m+1})$. Clearly, if $n = 0$ there is an extra zero singular value and the other singular values may be changed.

Theorem 5. *($m \to m+1$ Transition Theorem)*

1. $\sigma_{m+1}^{(m+1)} \leq v_{m+1}$.
2. $$\sigma_1^{(m+1)} \geq \sigma_1^{(m)} \geq \sigma_2^{(m+1)} \geq \ldots \geq \sigma_m^{(m+1)} \geq \sigma_m^{(m)} \geq \sigma_{m+1}^{(m+1)}.$$
3. *For* $i = 1, \ldots, m$
$$\sigma_i^{(m)} \leq \sigma_i^{(m+1)} \leq (1 + 2||\alpha||)\sigma_i^{(m)} + v_{m+1}$$
and
$$(\sigma_i^{(m)})^2 \leq (\sigma_i^{(m+1)})^2 \leq (1 + 2||\alpha||)(\sigma_i^{(m)})^2 + v_{m+1}^2$$
where $\alpha = (\alpha_1, \ldots \alpha_m)$.
4. *If* $v_{m+1} > 0$, $\mathrm{sdet} M_{m+1} = v_{m+1}\mathrm{sdet} M_m$.

Proof. To prove this result we introduce the matrices M' and M'' which are the same as M_m except that an extra row is added to the bottom of M_m. For M' this last row is equal to $\sum_{\ell=1}^m \alpha_\ell c_\ell$ and for M'' the row added is all zeros. Then $M' = AM''$ where A is the matrix with all entries zero except for ones down the diagonal and bottom row given by $(\alpha_1, \ldots, \alpha_m, 1)$. Clearly, the positive singular values of M_m and M'' are identical.

In order to compare the singular values of M' and M'' we need to estimate the norm of $A^t - A^{-1}$ which measures how far A is from being orthogonal. Since A^{-1} is obtained from A by replacing α_i by $-\alpha_i$ for $i = 1, \ldots, m$, then

$$A^t - A^{-1} = \begin{pmatrix} 0 & \cdots & 0 & \alpha_1 \\ \vdots & & \vdots & \vdots \\ 0 & \cdots & 0 & \alpha_m \\ \alpha_1 & \cdots & \alpha_m & 0 \end{pmatrix}.$$

Consequently, $||A^t - A^{-1}|| \leq 2||\alpha||$ where $\alpha = (\alpha_1, \ldots, \alpha_m)^t$.

Since $\sigma_1^{(m)} \geq \sigma_2^{(m)} \geq \cdots \geq \sigma_m^{(m)}$ and 0 are the singular values of M'', using Theorem 3.3 of [4] we deduce that if $\sigma_1' \geq \sigma_2' \geq \cdots \geq \sigma_m'$ are the first m singular values of M' then those that are non-zero satisfy

$$|\sigma_i' - \sigma_i^{(m)}| \leq ||A^t - A^{-1}||\sigma_i^{(m)} \leq 2||\alpha||\sigma_i^{(m)}. \qquad [12]$$

The other singular value is zero.

Now we relate v_{m+1} and the singular values of M' to those of M_{m+1}. We have $M_{m+1} - M' = N$ where N is the $m \times s$ matrix all of whose entries are zero except the last row which is the row vector n. Thus, $||N||_2 = ||n||$.

But, by Part (c) of Theorem 3.3.16 of [8]

$$|\sigma_i(M_{m+1}) - \sigma_i(M')| \leq \sigma_1(M_{m+1} - M') = \sigma_1(N) = ||N||_2. \qquad [13]$$

Combining this with (12) gives the required result for the first statement of part 3 and putting $i = m+1$ in equation (13) gives part 1 since $\sigma_{m+1}(M') = 0$. Part 4 of the theorem follows directly from theorem 1 and part 2 from the interlacing property for singular values (1). The second statement of Part 3 follows by applying the same steps as those used to prove the first statement except that instead of applying them to $M' = AM''$ and $M_{m+1} = M' + N$ one applies them respectively to $M'(M')^t = AM''(M'')^t A^t$ and $M_{m+1}M_{m+1}^t = M'(M')^t + NN^t$. QED

6. Constructing effective artificial likelihoods

Recall that we consider functions of the form

$$\varphi_m(k) = -\sum_{i=1}^m a_i^2 (C_i(k) - C_i^0)^2 \qquad [14]$$

We suppose that the C_i have been ordered so as to produce an ordered set of linearised constraints $c_1, \ldots, c_m$ and that we are adding a new constraint C_{m+1} with linearisation c_{m+1}. Using Theorem 5 we deduce that the smallest singular value of M_{m+1} is no greater than $v_{m+1} = \mathrm{val}(c_{m+1}|c_1, \ldots, c_m)$ and that its other singular values interlace with those of M_m as in Part 2 of Theorem 5 and satisfy Part 3 of Theorem 5. Since the singular values of the FIM F_{m+1} are the squares of those for M_{m+1} we have the required result.

7. Constraint calculation and ranking

All the constraints can easily be calculated using SASSy: Sensitivity Analysis Software of Systems available from: http://www2.warwick.ac.uk/fac/sci/systemsbiology/research/software/. In order to rank the constraints, we use the qr function from Matlab [12] and perform the QR decomposition on the matrix M^t. Additional option for column-pivoting allows us to rank the entries along the main diagonal of the matrix $L = R^t$ in order of decreasing value. These entries along the diagonal of L are the constraint values, v_j.

8. Pokhilko model

First target of the parameter optimization performed in [19] is the fit to normalized data of eight mRNA time series in WT plant in diurnal light conditions of 12h light and 12h darkness (12L:12D). The fit to mRNA levels give rise to 82 linearized constraints of type shown in Equation 8 in the main text. In Supplementary Table 1 we give a breakdown of the number of constraints coming from each mRNA level measurement, as well as list the data that they are based on. Most of the time series data (but not the SEs) used to constrain the model can be found at: http://www.amillar.org. Aside from fitting the model to the time series, the Pokhilko clock model is also fitted to period constraints. The period estimates are outlined in the Main Text.

We scale the constraints based on timeseries measurements by standard errors (SE) of the timeseries that we obtain from the literature (see Supplementary Table 1). Since the model in [19] is fitted to timeseries that have been normalized (by dividing them by their maximum values), the SEs are scaled by the same factor as the timeseries. These SE and SD come from the literature with specific data sources listed in Table 1. Below we outline the details of the scaling.

For some of the timeseries used there were no standard errors provided. In these cases we did the following:

1. In the case the whole timeseries has some SEs, for the points with no SEs, we assign a value of the error of the point with the closest mean value (for which the SE exists). We followed this approach when assigning SEs for LHY measurements (taken from [5]), as well as , ELF3, LUX, ELF4, PRR9 (from [7]) and GI measurements (from [9]).
2. In some cases, the constraints for a particular gene are based on several timeseries and some of these have no SEs. For these cases, we selected the SEs from the timeseries that did have them, following the approach described in the previous point. This selection had to be done for constraints on PRR9 levels based on data from [16]and GI levels based on data from [10]. Constraints for LHY/CCA1 component are based on timeseries of two genes: LHY (from [5]) and CCA1 (from [3]).We only take the SEs from [5] and give the same SEs to the constraint taken from [3], following the approach described above.
3. In the case the constraint is based on gene timeseries with no SEs, we take the SEs of a timeseries of another gene, if the non-normalized levels of the timeseries are similar. This approach had to be taken when assigning SEs to PRR7 and TOC1 levels coming from measurements of [16]. Data from [16], that is, PRR9 , PRR7 and TOC1 levels measured by Northern blots, have no SEs, but the levels of all three are similar and we could used the SEs from another measurement of PRR9 (from [7]) to assign the missing SEs.

We scale the period constraints by either standard deviations (SDs) or SEs associated to each period measurement, depending on which is available in the literature. For period estimates in WT under LL and *toc1* mutant backgrounds, SDs are taken from [14], while those for periods in *prr7/9* and *ztl* mutant backgrounds are taken from RAE plots from [5] and [13], respectively.

To perform parameter perturbations, from the original parameter set, P_1 (from [19]) we simulated 25 new parameter sets with each parameter k_j allowed to vary from it's original value by δk_j where $\delta k_j \in \mathcal{N}(0,(0.05k_j)^2)$. From these 25 sets, we discarded 12 sets that could not maintain oscillations in all four mutant backgrounds, since their set of constraints could not include some of the period constraints which means that they are not directly comparable the the original model. From the remaining ones, we selected 10 sets where the model solutions for the relevant mRNAs (i.e. constraints from the κ_1 background) and periods (constraints from all the other backgrounds) were closest to the solutions and periods of the original (i.e. P_1 set) model. As a measure of closeness, we aimed to minimize the sum of the residual sum of squares (RSS) of solutions added to the RSS of the periods.

9. Locke model

Constraints placed by Locke et al. are outlined in detail in [11]. Assuming that for the model parameter set k_* the constraints placed by Locke et al. are already achieved, any small enough changes to parameter values will not affect some of these constraints. For example, for such small parameter changes, model will still be able to entrain to 24h light-dark cycles (if it could do so for parameter set k_*) and its oscillations will remain large enough to be detected, even if their actual amplitude may vary. Full list of GE-combinations and constraints that are translated to our framework are provided in the Supplementary Table 3.

We give a brief description of how the constraint for time of peak (or trough), Equation 6 (in the main text) is written in terms of the partial derivative $\partial g_{j,\kappa}/\partial k$. Let ϕ be a time of peak (or trough) of solution $g_\kappa(t,k)$ for its j-th variable. Here $g_\kappa(t,k)$ is a solution of ODE system given in Equation 1 in the main text. Constraint for keeping time of peak (or trough) fixed is,

$$c(k) = \frac{\partial \phi}{\partial k}.$$

The above derivative can be written as

$$\frac{\partial \phi}{\partial k} = \frac{1}{\ddot{g}_{j,\kappa}(\phi)}\left(J(\phi)_j\frac{\partial g_\kappa}{\partial k}(\phi) + \frac{\partial f_{j,\kappa}}{\partial k}(\phi)\right) \qquad [15]$$

with $J(\phi)_j$ as the j-th row of the Jacobian matrix evaluated at the time ϕ. Mathematical derivation of Equation 15 is given in [20].

Amplitude, $A(k)$, of the oscillation is a difference in the value of solution $g_\kappa(t,k)$ at two time-points indicating the peak and trough, ϕ_1 and ϕ_2, respectively. Constraint on amplitude is,

$$c(k) = \frac{\partial g_\kappa}{\partial k}(\phi_1) - \frac{\partial g_\kappa}{\partial k}(\phi_2).$$

The amplitude of oscillations of various components can be of several magnitudes different, so we consider constraining $log(A(k))$. This requires just multiplying the above equation by a factor $1/A(k_*)$.

Models of the *toc1* mutant plant (in 12L:12D and constant light LL conditions) have multiple non-functioning subnetworks. Aside from TOC1 protein (cytoplasmic and nuclear), X gene components (mRNA, and cytoplasmic and nuclear proteins) also converge to zero, since transcription of X mRNA depends solely on TOC1 nuclear protein level. Since in the mutant model the translation rate of TOC1 is set to zero and is not allowed to change, the levels of TOC1 protein and X mRNA and protein, will remain at zero independently of other perturbations made to the parameters. Hence, constraints will not depend on parameters coming from the five ODEs representing TOC1 proteins, and X mRNA and proteins. Thus, we ignore these parameters and set their constraint entries to zero. TOC1 and X also have effect on LHY and Y mRNAs through transcription, but since mutant model levels of TOC1 and Y proteins are zero, parameters of a term describing LHY

transcription via X nuclear protein, and a term describing Y transcription via TOC1 nuclear protein will have no effect on models dynamics. Thus we can also ignore the effects of these parameters viz. n_1, g_1, a, e and g_5, by setting the associated entries of linearised constraints to zero.

Again, the constraints are scaled by the error measurements of the desired feature. The time series data presented in [11] does not have standard error measurements. Below we illustrate how we determined the errors for the WT model features:

1. For peak and trough measurements, since the measurements are at 2h intervals, we assume that SE is half the time interval, so $SE = 1h$.
2. For estimates of amplitude, sharpness of peak and broadness of trough of LHY/CCA1 mRNA, we take the similar LHY mRNA measurements from [5] and normalize them (and their errors) by the maximum value. Measurements from [5] are taken every 4h, and many of the SEs are not visible from the graphics (especially those close to trough values). Hence, we only take the scaled SE of the LHY peak value (located at dawn time), with the value $SE = 0.1860$, to be the standard error associated to all these other qualitative features.

For other the models in other backgrounds (i.e. *toc1* and *lhy/cca1* mutant models under LD conditions), we also take the errors to be identical to those of the WT system (as detailed above).

We scaled the period constraints by the error measurements (SDs) for periods that we obtain from the literature. SDs of period estimates for WT in LL and DD are $SD = 0.4h$ [14] and $SD = 1.1h$ [22], respectively. Period estimate of *toc1* mutant in LL conditions is $SD = 0.6h$ [14]. We have been unable to locate in the literature the error estimates for the *lhy/cca1* period measurements, but since the period value is comparable to that of *toc1* mutant in LL, we also take the error to be $SD = 0.6h$.

10. NF-κB model

Since N:C NF-κB does not appear as a variable of the NF-κB model of [1], for our analysis, we have to introduce an ODE for $\frac{d}{dt}$(N:C NF-κB)(t). This ODE can be obtained by differentiating with respect to time the expression

$$(\text{N:C NF-}\kappa\text{B})(t) = \frac{1}{k_v}\frac{(n\text{NF-}\kappa\text{B}_{tot})(t)}{(c\text{NF-}\kappa\text{B}_{tot})(t)} \quad [16]$$

where k_v is the a spatial parameter of the measured C:N ratio and nNF-κB$_{tot}(t)$ and cNF-κB$_{tot}(t)$ are total concentrations of nuclear and cytoplasmic NF-κB. Total concentration of nuclear NF-κB is a sum

$$(n\text{NF-}\kappa\text{B}_{tot})(t) = (\text{nNF-}\kappa\text{B})(t) + (\text{nI}\kappa\text{B}\alpha \circ \text{NF-}\kappa\text{B})(t)$$

where nNF-κB and nIκB$\alpha \circ$ NF-κB are variables describing nuclear concentration of free and IκBα-bound NF-κB, respectively. Total concentration of cytoplasmic NF-κB is

$$\begin{aligned}(c\text{NF-}\kappa\text{B}_{tot})(t) \quad = \quad & (\text{NF-}\kappa\text{B})(\text{t}) + (\text{I}\kappa\text{B}\alpha \circ \text{NF-}\kappa\text{B})(\text{t}) \\ & +(\text{pI}\kappa\text{B}\alpha \circ \text{NF-}\kappa\text{B})(\text{t})\end{aligned}$$

where NF-κB IκB$\alpha \circ$ NF-κB and pIκB$\alpha \circ$ NF-κB describe free, unphosphorylated and phosphorylated IκBα -bound NF-κB in the cytoplasm, respectively.

Full list of constraints used to parametrize the NF-κB model are given in [1], Table S5 in SI. Features that do not need to be translated to our framework are the following. Ashall et al. state that the initial (equilibrium) level of N:C NF-κB as TNF-α is introduced must be in the range of 0.05 ± 0.04(SD). For the model with unperturbed parameter values, the initial concentration of N:C NF-κB is within the stated range. Since for a small enough perturbation of parameters, the perturbed equilibrium value will still remain within the stated range, the target of [1] will be automatically satisfied and hence it needs not to be added to our list of linearised constraints.

Ashall et al. identify four key features for the N:C NF-κB subject to a continuous TNF-α signal. First feature is that N:C NF-κB must peak at least five times within the 600 minutes interval from the start of the TNF-α input. Under the assumption that for the model parameters values all peaks and troughs identified are quadratic, this target will be satisfied by any small enough perturbation of the parameters. Second feature of the model is that the troughs of N:C NF-κB oscillations must be higher than the initial $t = 0$ level of N:C NF-κB . The model already satisfies this condition and any small enough perturbation in the parameters can be made so that this feature is preserved. Hence, it also does not need to be added to our list.

The list of constraints from Ashall et al. that have to be translated to our framework are given in Supplementary Table 5. The constraints are based on four GE-combinations.

We now review the construction of the constraints. Unlike in the Pokhilko model, in the NF-κB model the levels of N:C NF-κB peaks can vary by fold of four (compare for example the expected peaks in constant TNF-α vs. first peak in TNF-α pulse experiments). So, for any solution $g_\kappa(\phi)$ coming from some GE-combination κ and evaluated at peak time ϕ, we consider a constraint on the peak level of the type

$$c(k) = \left(\frac{1}{g_\kappa(\phi, k_*)}\right) \cdot \frac{\partial g_\kappa(\phi, k_*)}{\partial k}$$

where we have non-dimensionalised and normalized the level of NF-κB by scaling by $1/g_\kappa(\phi, k_*)$.

Constraints for keeping times of peaks fixed are constructed from Equation 8 that we identified in the main text. Constraint for peak-peak time between two peaks (in GE-combination κ_1, Table 5) is just a difference of two peak time derivatives,

$$c(k) = \frac{\partial \phi_1}{\partial k} - \frac{\partial \phi_2}{\partial k}$$

where ϕ_1 and ϕ_2 denote the peak times. Constraints for fixing the ratio of peaks (1st to 2nd or 1st to 3rd) follow from Equation 9, shown in the main text.

The 25 linearised constraints of NF-κB model are compiled into an 25×28 matrix M. We find that columns of M corresponding to parameters v_t and $k1ta$ are columns of zeros. These two parameters do not affect N:C NF-κB dynamics. This is not surprising since, t_v, describing the volume size, is not featured in the model equations, and parameter $k1ta$ describes degradation of a component, phosphorylated IκBα, that does not affect any other variables of the model, including N:C NF-κB.

As with the previous models, the constraints are scaled by the errors of the desired feature measurements. NF-κB model of [1] is fit using specific cost functions (described in Table S5, [1]) where a score of 1 is set to approximately match one standard deviation from the mean of the respective feature that is matched by the model. We use the cost functions in order to give error estimates. For the model in continuous TNF-α background, we take the standard deviation of the peak levels to be $SD = \sqrt{3}$min, while phase time and peak-to peak times

have errors $SD = \sqrt{600}$min. For models under 60, 100 and 200 minute repeat TNF-α pulses, there are no peak timing constraints (but these do form constraints in our framework), so we take standard deviations of phases as $SD = \sqrt{600}$min. Error of first peak value is taken as $SD = \sqrt{3}$ and other ratios of peak values are taken as having error $SD = \sqrt{6}/10$.

11. Ubiquity of small norms

We illustrate the argument of section 4 of the main paper about why one expects to get small norms. It is argued there that the $\tilde{D}_i = (d_i \cdot U_1, \ldots, d_i \cdot U_s)$ can be regarded as a random vector of norm $\leq$ O(1). It follows from the argument below that for $k \ll s$,

$$(d_i \cdot U_1)^2 + \cdots + (d_i \cdot U_k)^2 \leq \varepsilon$$

with probability $p(s) = 1 - \mathrm{O}(e^{-\varepsilon s/4})$. It follows that, with this probability, if $c_i = \tilde{D}_i \cdot W$, then

$$||c_i||^2 = ||\tilde{D}_i||^2 \leq \tilde{\sigma}_1^2 \varepsilon + \mathrm{O}(\tilde{\sigma}_{k+1}^2)$$

since W is orthogonal.

Let $g_1, \ldots, g_s$ be independent. mean zero, Gaussian variable with unit variance and let μ be normalised Haar measure on the unit sphere $\mathbb{S}^{s-1}$. Then it is well-known that, by the invariance of the canonical Gaussian measure under orthogonal transformations, $||g||^{-1}(g_1, \ldots, g_s)$ has the same distribution as $a = (a_1, \ldots, a_s)$ does with respect to $d\mu(a)$. We estimate an upper bound for $\mu(a_1^2 + \cdots + a_k^2 > 2C)$ as follows:

$$\begin{aligned}\mu(a_1^2 + \cdots + a_k^2 > 2C) &= P(\frac{g_1^2}{||g||^2} + \cdots + \frac{g_k^2}{||g||^2} > 2C) \\ &= P(g_1^2 + \cdots + g_k^2 \geq 2C||g||^2) \\ &\leq P(g_1^2 + \cdots + g_k^2 \geq Cs) \\ &\quad + P(||g||^2 < s/2).\end{aligned}$$

The last inequality is from the split between the cases $||g||^2 \geq s/2$ and $||g||^2 < s/2$.

Now $g_1^2 + \cdots + g_k^2$ and $||g||^2$ are chi-squared distributed with k and s degrees of freedom respectively. When there are K degrees of freedom, the cumulative distribution of the chi-squared distribution is $F(K/2, x/2)$ where F is the regularized Gamma function (e.g. [21]). If $z = x/K < 1$ then $F(K/2, x/2) \leq (ze^{1-z})^{K/2}$. Therefore,

$$P(||g||^2 < s/2) \leq (\frac{1}{2}e^{1/2})^{s/2}) \approx (0.824\ldots)^{s/2}.$$

If $z = x/K > 1$ then $1 - F(K/2, x/2) \leq (ze^{1-z})^{k/2}$. Thus,

$$\begin{aligned}P(g_1^2 + \cdots + g_k^2 \geq Cs) &= 1 - F(k/2, Cs/2) \\ &\leq \left(\frac{Cse}{k}\right)^{k/2} e^{-Cs/2}. \qquad [17]\end{aligned}$$

1. Ashall L et al. (2009) Pulsatile stimulation determines timing and specificity of NF-kappa B-dependent transcription. Science 324: 242–246.
2. Dixon L et al. (2011) Temporal repression of core circadian genes is mediated through early Flowering 3 in arabidopsis. Curr Biol 21:120–125.
3. Edwards K-D et al. (2010) Quantitative analysis of regulatory flexibility under changing environmental conditions. Mol Syst Biol 6: 424.
4. Eisenstat, S C & Ipsen, ICF. (1995) Relative perturbation techniques for singular value problems. SIAM Journal on Numerical Analysis 32(6) 1972-1988
5. Farre E, Harmer A, Harmon F, Yanovsky M, and Kay S. (2005) Overlapping and distinct roles of prr7 and prr9 in the arabidopsis circadian clock. Curr Biol, 15:47–54.
6. Hazen S et al. (2005) Lux arrhythmo encodes a myb domain protein essential for circadian rhythms. Proc Natl Acad Sci USA, 102:10387–10392.
7. Helfer A et al. (2011) Lux arrhythmo encodes a nighttime repressor of circadian gene expression in the arabidopsis core clock. Curr Biol, 21:126–133.
8. Horn, R. A. & Johnson, C. R. Topics in Matrix Analysis. (1991) Cambridge University Press.
9. Kim W et al. (2007) Zeitlupe is a circadian photoreceptor stabilized by gigantea in blue light. Nature, 449:365–360.
10. Locke J-W-C et al. (2005) Extension of a genetic network model by iterative experimentation and mathematical analysis. Mol Syst Biol 1: 2005.0013
11. Locke J-W-C et al. (2006) Experimental validation of a predicted feedback loop in the multi-oscillator clock of Arabidopsis Thaliana. Mol Syst Biol 59: -.
12. MATLAB 7.11 (R2010b), The MathWorks, Inc., Natick, Massachusetts, United States.
13. Mas P, Kim WY, Somers DE, Kay SA (2003) Targeted degradation of TOC1 by ZTL modulates circadian function in Arabidopsis thaliana. Nature 426: 567–570
14. Millar AJ, Carre IA, Strayer CA, Chua NH, Kay SA (1995) Circadian clock mutants in Arabidopsis identified by luciferase imaging. Science 267: 1161–1163.
15. McWatters H et al. (2007), Elf4 is required for oscillatory properties of the circadian clock. Plant Physiol, 144:391–401.
16. Nakamichi N, Matsushika A, Yamashino T, and Mizuno T. (2003) Cell autonomous circadian waves of the aprr1/toc1 quintet in an established cell line of arabidopsis thaliana.Plant Cell Physiol, 44:360–365.
17. Nakamichi N et al. (2005)The arabidopsis pseudo-response regulators, prr5 and prr7, coordinately play essential roles for circadian clock function. Plant Cell Physiol, 46:609619.
18. Numerical Recipes: The Art of Scientific Computing. Third Edition (2007), 1256 pp. Cambridge University Press ISBN-10: 0521880688
19. Pokhilko A et al. (2012) The clock gene circuit in arabidopsis includes a repressilator with additional feedback loops. Mol Syst Biol 8.
20. Rand D-A (2008) Mapping the global sensitivity of cellular network dynamics: Sensitivity heat maps and a global summation law. J. R. Soc. Interface, 5: S59-S69.
21. Simon, M. K. (2002) Probability Distributions Involving Gaussian Random Variables. Springer (New York) ISBN 978-0-387-34657-1
22. Strayer C, Oyama T, Schultz TF, Raman R, Somers DE, Mas P, Panda S, Kreps JA, Kay SA (2000) Cloning of the Arabidopsis clock gene TOC1, an autoregulatory response regulator homolog. Science 289: 768–771

Figures

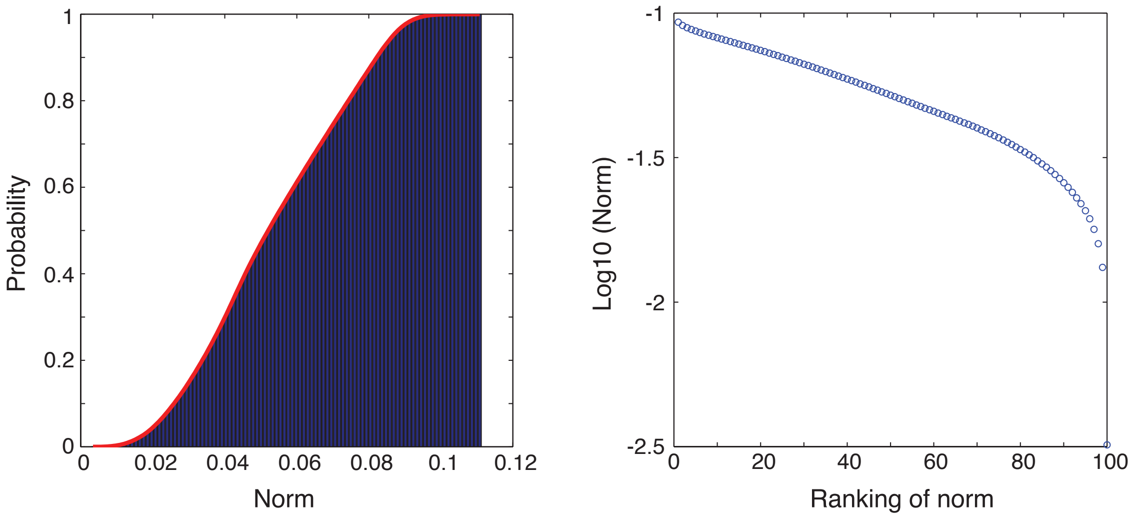

Fig. 1. (Left) the cumulative distribution of the norms of vectors of the form $(\sigma_1 v_1, \ldots, \sigma_s v_s)$ where s is large, $(v_1, \ldots, v_s)$ is a random vector and $\sigma_i = 2^{-i}$. (Right) The asymptotic shape of the curve obtained when one ranks $N = 100$ such norms draw randomly from the pdf associated with the CDF. As explained in the main text the ith values is given by $x_i = G^{-1}(1 - i/N)$ where G is the CDF.

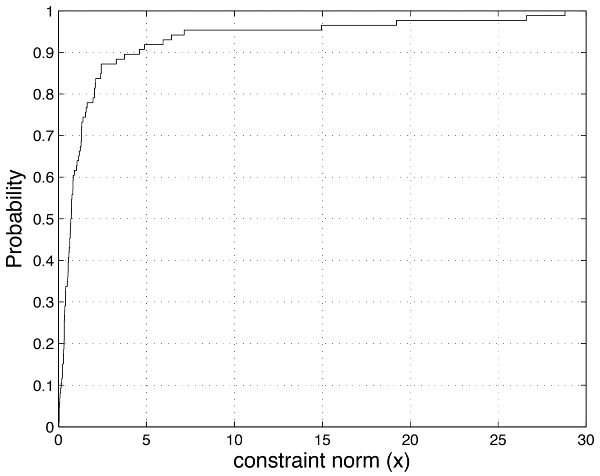

Fig. 2. The cumulative distribution of the unnormalized norms of the Pokhilko model.

Tables

Table 1. Breakdown of the constraints for GE-combination κ_1 in the Pokhilko model [19]

mRNA	Data source	No. of constraints
TOC1	Nakamichi et al. [17]	8
PRR9	Helfer et al. [7]; Nakamichi et al.[16]	11
PRR9	Nakamichi et al. [16]	9
GI	Kim et al. [9]; Locke et al. [10]	18
ELF3	Dixon et al. [2]	13
ELF4	McWatters et al. [15]	6
LUX	Hazen et al. [6]; Helfer at al. [7]	6
LHY/CCA1	Edwards et al. [3]; Farre et al. [5]	13

Table 2. Top 20 ranked constraints of the Pokhilko 2012 model

Ranking	Constraint	GE-combination
1	GI level 13h after dawn	WT 12L:12D
2	period	*ztl* mutant
3	GI level 10.4h after dawn	WT 12L:12D
4	period	WT in constant light
5	PRR7 level 13.8h after dawn	WT, 12L:12D
6	PRR9 level 1.29h after dawn	WT, 12L:12D
7	LHY level 20h after dawn	*ztl* mutant
8	GI level 5h after dawn	WT, 12L:12D
9	TOC1 level 15.4h after dawn	WT, 12L:12D
10	LHY level 4h after dawn	WT, 12L:12D
11	period	*prr7/prr9* mutant
12	LUX level 12h after dawn	WT, 12L:12D
13	ELF3 level 0h after dawn	WT, 12:12D
14	PRR7 level 19.4h after dawn	WT, 12L:12D
15	PRR9 level 4h after dawn	WT, 12L:12D
16	PRR7 level 7.3h after dawn	WT, 12L:12D
17	GI level 0.74h after dawn	WT, 12L:12D
18	TOC1 level 12.3h after dawn	WT, 12L:12D
19	GI level 15.38h after dawn	WT, 12L:12D
20	period	*toc1* mutant

Table 3. The GE combinations of the Locke model [11].

GE comb.	Genetic background	Entraining signal	Constraint description	No. of constraints (per GE-combination)
κ_1	WT	12L:12D	peak times of LHY mRNA and TOC1 mRNA;<br>trough times of LHY mRNA and TOC1 mRNA;<br>amplitude of oscillations of LHY mRNA and TOC1 mRNA;<br>sharp peak of LHY mRNA;<br>broad trough of LHY mRNA	8
κ_2	WT	LL	period	1
κ_3	WT	DD	period	1
κ_4	*toc1*	12L:12D	peak times of LHY mRNA and PRR7/9 mRNA;<br>amplitude of oscillations of LHY mRNA and PRR7/9 mRNA;<br>sharp peak of LHY mRNA;	5
κ_5	*toc1*	LL	period	1
κ_6	*lhy/cca1*	12L:12D	peak times of TOC1 and Y mRNA;<br>amplitude of oscillations of TOC1 and Y mRNA;<br>sharp peak of Y mRNA;	6
κ_7	*lhy/cca1*	DD	period	1
κ_8	*prr7;prr9*	LL	period (not-included as no long term rhythm)	N/A

February 2, 2015

Table 4. Top 17 ranked constraints of the Locke 2006 model

Ranking	Constraint	GE-combination
1	period	WT, LL
2	LHY amplitude	WT, 12L:12D
3	period	*lhy/cca1*, DD
4	rate of LHY level fall after peak	*toc1*, 12L:12D
5	period	*toc1*, LL
6	TOC1 amplitude	WT, 12L:12D
7	TOC1 peak time	WT, 12L:12D
8	LHY trough	WT, 12L:12D
9	Y amplitude	*lhy/cca1* mutant, 12L:12D
10	PRR7/9 amplitude	*toc1* mutant, 12L:12D
11	TOC1 amplitude	*lhy/cca1* mutant, 12L:12D
12	TOC1 peak time	*lhy/cca1*, 12L:12D
13	LHY peak difference from 2h after peak vs at peak	WT, 12L:12D
14	LHY peak difference from 2h before peak vs at peak	WT, 12L:12D
15	Y peak time	*lhy/cca1*, 12L:12D
16	PRR7/9 amplitude	*toc1*, LL
17	period	WT, DD

Table 5. The GE combinations of the NF-κB model [1]

GE comb.	Entraining signal	Constraint descriptions	No. of constraints (per GE-combination)
κ_1	constant	peak values of 3rd, 4th and 5th peak<br>peak times of 3rd, 4th and 5th peak<br>peak-peak time of 2nd to 3rd peak	7
κ_2	60 min repeat of 5 min pulses	peak times of 1st, 2nd and 3rd peak<br>peak value of 1st peak<br>ratio of values of 1st and 2nd peak<br>ratio of values of 1st and 3nd peak	6
κ_3	100 min repeat of 5 min pulses	peak times of 1st, 2nd and 3rd peak<br>peak value of 1st peak<br>ratio of values of 1st and 2nd peak<br>ratio of values of 1st and 3nd peak	6
κ_4	200 min repeat of 5 min pulses	peak times of 1st, 2nd and 3rd peak<br>peak value of 1st peak<br>ratio of 1st to 2nd peak level<br>ratio of values of 1st and 3nd peak	6
